# Mechanism of Pingchuanning in Attenuating Asthma Based on UHPLC‐Q‐Orbitrap HRMS and Network Pharmacology Analysis

**DOI:** 10.1002/bmc.70607

**Published:** 2026-10-19

**Authors:** Yue Zhang, Yiwen Ding, Weidong Chen, Yuanbo Bi, Yanyan Wang, Weidong Ye, Xiangming Fang

**Affiliations:** ^1^ Anhui University of Chinese Medicine Hefei China; ^2^ School of Traditional Chinese Medicine Anhui University of Chinese Medicine Hefei China; ^3^ School of Pharmacy Anhui University of Chinese Medicine Hefei China; ^4^ Key Laboratory of Xin'an Medicine, Ministry of Education Anhui University of Chinese Medicine Hefei China

**Keywords:** ADORA2A, asthma, network pharmacology, Pingchuanning, UHPLC‐Q‐Orbitrap HRMS

## Abstract

An ultrahigh‐performance liquid chromatography coupled with quadrupole‐Orbitrap high‐resolution mass spectrometry (UHPLC‐Q‐Orbitrap HRMS) method was applied to characterize Pingchuanning (PCN) decoction and its absorbed serum constituents in asthmatic rats. A total of 107 compounds were identified in PCN, of which 36 prototypes were detected in rat serum. Network pharmacology and molecular docking of these absorbed compounds predicted ADORA2A as the core therapeutic target, with 5‐*O*‐methylvisammioside, nobiletin, *sec*‐*O*‐glucosylhamaudol, and 9‐oxo‐ODE showing binding affinity. In an ovalbumin‐induced asthmatic rat model, PCN alleviated airway hyperresponsiveness and lung inflammation. Mechanistically, PCN upregulated ADORA2A, elevated cAMP and PKA phosphorylation, and suppressed NLRP3 expression. This UHPLC‐Q‐Orbitrap HRMS‐based serum pharmacochemistry method effectively identified the bioactive components of PCN, and the integrated strategy demonstrates that PCN exerts anti‐asthmatic effects through the ADORA2A/cAMP/PKA/NLRP3 axis.

## 1 Introduction

Asthma is a common long‐term inflammatory condition that affects the airways. Its main features are nonspecific inflammation and hyperresponsiveness of the bronchial tubes(C. Liu, Zeng, et al. 2025). The condition typically presents with repeated wheezing, shortness of breath, chest tightness, and cough as its main clinical features (Hammad et al. 2025). Current standard therapies are effective in managing acute symptoms but are limited by adherence issues and risks associated with overuse of short‐acting β_2_‐agonists (Jayasooriya et al. 2025; Miller et al. 2021).

In traditional Chinese medicine (TCM) theory, asthma is attributed to constitutional weakness or acquired fatigue and internal injury, leading to dysfunction of the zang‐fu organs, airway obstruction, and failure of the lung to disperse and descend qi (X. H. Wang, Shao, et al. 2025). These pathological changes manifest clinically as tachypnea and audible wheezing (Polverino and Sin 2026). Grounded in holistic regulation and syndrome differentiation, TCM has demonstrated unique advantages in asthma management (Y. Liu, Wu, et al. 2025). Accumulating evidence indicates that both herbal formulas (Xu et al. 2026) and their active components (Jia et al. 2022) can significantly alleviate clinical symptoms and airway inflammation in asthmatic patients. Pingchuanning (PCN), a modified formulation derived from the classic prescriptions Shegan Mahuang Decoction and Sanzi Yangqin Decoction, comprises 11 herbal ingredients, including Ephedrae herba, Saposhnikoviae radix, and Fritillariae thunbergii bulbus. It is recognized for its effects in warming the lung, resolving phlegm, relieving cough, and suppressing wheezing, with notable therapeutic efficacy and well‐defined actions (Fang et al. 2012; Wang et al. 2019).

Chronic airway inflammation constitutes a central mechanism in asthma pathogenesis (Joulia et al. 2025). Accompanying pulmonary inflammation in asthmatic rats is the activation of the NLRP3 (NOD‐like receptor protein 3) inflammasome—a major initiator of asthmatic airway inflammation (DeStefano et al. 2025). Through the adaptor protein ASC (apoptosis‐associated speck‐like protein with a CARD), NLRP3 drives the activation of caspase‐1. This activation triggers pyroptosis and also facilitates the secretion of two pro‐inflammatory cytokines, IL‐1β and IL‐18. Through these events, airway inflammation becomes sustained (Yang et al. 2024). Recent studies have shown that ADORA2A promotes Tfh cell differentiation and Type 2 airway inflammation through the AKT signaling pathway. Regarding NLRP3, clinical evidence demonstrates that increased ability for inflammasome priming and activation is a common feature of systemic immune cells in both severe and non‐severe asthma, with MCC950 effectively suppressing IL‐1β release {Jo, 2016 #172} {Kodi, 2024 #173}. Moreover, NLRP3 expression is upregulated in induced sputum of patients with asthma and correlates with disease severity. However, the bioactive constituents of PCN and the precise molecular mechanisms through which it regulates NLRP3 remain unclear.

In this study, we aim to characterize the chemical profile of PCN and identify its absorbed components in asthmatic rats using UHPLC‐Q‐Orbitrap HRMS. A combination of network pharmacology and molecular docking will be utilized to screen for prospective active compounds and their cognate targets. The predicted mechanisms will subsequently be validated through in vivo experiments. Collectively, this study seeks to elucidate the bioactive basis of PCN and the mechanisms by which it modulates NLRP3 in the treatment of asthma.

## 2 Methods

### 2.1 Chemical and Reagents

The crude herbal materials used in PCN—including Ephedrae herba, Asari radix et Rhizoma, Armeniacae semen amarum, Perillae fructus, Citri reticulatae pericarpium, Fritillariae thunbergii bulbus, Astragali radix, Pseudostellariae radix, Pinelliae rhizoma, Pheretima, and Saposhnikoviae radix—were purchased from the Guoyitang of Anhui University of Chinese Medicine and authenticated by experienced pharmacognosists based on morphological characteristics. Voucher specimens have been deposited at the Herbarium of Anhui University of Chinese Medicine. The PCN decoction was prepared following the method established in our previous study.

Ovalbumin (OVA) (Batch No. 0000422880) and aluminum hydroxide adjuvant (Batch No. MKW2481) were obtained from Sigma‐Aldrich. Dexamethasone (Dex) was sourced from SPH Sine (Shanghai, China). Acetyl‐β‐methylcholine chloride (ACh) (Batch No. KS396521) and reference standards including asarinin (Batch No. J281B218756), amygdalin (Batch No. Y08O10K96808), prim‐*O*‐glucosylcimifugin (Batch No. S25GB162230), rosmarinic acid (Batch No. JB241724), calycosin‐7‐*O*‐β‐D‐glucoside (Batch No. JB238425), 5‐*O*‐methylvisammioside (Batch No. J23HB186177), hesperidin (Batch No. JB269789), tangeretin (Batch No. J251B218072), astragaloside IV (Batch No. N13HB201030), and nobiletin (Batch No. S161B226120) were also obtained from Shanghai Yuanye Bio‐Technology Co. Ltd., all with mass fractions of ≥ 98.0%. Methanol and acetonitrile (both of mass spectrometry grade) were purchased from Merck, Germany, and formic acid was purchased from Sigma Aldrich, USA. Distilled water was obtained from Shenzhen Watson's Distilled Water Co. Ltd.

### 2.2 Qualitative Analysis of PCN's Chemical Compounds by UPLC‐Q‐Orbitrap MS

#### 2.2.1 Operating Conditions of the UPLC‐Q‐Orbitrap MS System

UPLC‐MS analysis was carried out using a Q Exactive Orbitrap mass spectrometer coupled with an UltiMate 3000 Rapid Separation Binary System (Thermo Fisher Scientific, USA). A HESI probe was mounted on the mass spectrometer, and chromatographic separation was carried out on an ACQUITY BEH C18 column (2.1 × 100 mm, 1.7 μm). The following parameters were used for both LC and MS analyses: Acetonitrile (A) and 0.1% formic acid in water (B) were used as mobile phases. The gradient elution program was 0–15 min, 95%–85% A; 15–25 min, 85%–74.4% A; 25–33 min, 74.4%–62.8% A; 33–48 min, 62.8%–38% A; 48–55 min, 38%–15% A; 55–60 min, 15%–95% A. A flow rate of 0.2 mL/min and an injection volume of 2 μL were applied, with the column temperature held at 30°C. For the HESI source, parameters were set as follows: sheath gas flow rate, 35 Arb; auxiliary gas flow rate, 15 Arb; sweep gas flow rate, 0 Arb; capillary temperature, 320°C; heater temperature, 275°C; spray voltage, 3.5 kV (positive mode), and 2.5 kV (negative mode). Data acquisition was performed in full MS/dd‐MS^2^ mode with the following settings: full MS resolution, 60,000 FWHM; MS/MS resolution, 30,000 FWHM; stepped collision energies, 20, 40, and 60 eV; mass range, m/z 100–1500. To ensure the reliability of identifying blood‐absorbed components, both blank serum and PCN‐dosed serum samples were analyzed under identical conditions. The total ion chromatograms (TICs) of the blank and dosed sera were compared to visually confirm the presence of newly appearing peaks derived from PCN. Data were processed with Compound Discover 3.3 and Xcalibur 4.0.

#### 2.2.2 Sample Solution Preparation

The accurately weighed herbal materials—comprising Ephedrae herba (9 g), Asari radix et rhizoma (3 g), Armeniacae semen amarum (9 g), Perillae fructus (6 g), Citri reticulatae pericarpium (6 g), Fritillariae thunbergii bulbus (9 g), Astragali radix (9 g), Pseudostellariae radix (9 g), Pinelliae rhizoma (9 g), Pheretima (6 g), and Saposhnikoviae radix (6 g)—were combined, amounting to a total of 81 g. The mixture was immersed in distilled water at a material‐to‐solvent ratio of 1:10 (g/mL) for 1 h, brought to a boil with vigorous heating, and then gently simmered for 45 min. This decoction was processed twice. The resulting extracts were combined and concentrated.

A mixed standard solution was prepared by combining accurately weighed portions of nine compounds (asarinin, amygdalin, rosmarinic acid, calycosin‐7‐*O*‐β‐D‐glucoside, tangeretin, hesperidin, nobiletin, 5‐*O*‐methylvisammioside, and prim‐*O*‐glucosylcimifugin). After dilution with methanol to 250 μg/mL, the mixture was filtered through a 0.22‐μm membrane. The obtained solution served as the mixed standard reference.

### 2.3 Establishment of a Network Pharmacology Framework

#### 2.3.1 Target Prediction for PCN‐Derived Active Components That Enter the Bloodstream

The SMILES notations for each compound were obtained from the PubChem database following the characterization of blood‐absorbed active ingredients in PCN by UHPLC‐Q‐Exactive HRMS. For those constituents that could not be retrieved from the database, their chemical structures were drawn using ChemDraw software, exported in MOL format, and subsequently converted to SMILES notations via the Open Babel toolkit. After obtaining the SMILES strings, we fed them into the SwissTargetPrediction database in batches (species restricted to *Homo sapiens* ), and predicted targets with a probability score > 0.1 were retained for further analysis. This step was done to predict potential targets. The resulting data were subsequently integrated and deduplicated to establish a comprehensive dataset of potential targets for PCN.

#### 2.3.2 Asthma‐Related Target Filtering and Cross‐Target Acquisition

Systematic searches were performed across five major disease databases, namely, GeneCards (https://www.genecards.org/), DisGeNET (https://www.disgenet.org/), DrugBank (https://go.drugbank.com/), PharmGKB (https://www.pharmgkb.org/), OMIM (www.omim.org), and TTD (https://db.idrblab.net/ttd/), using “Asthma” as the keyword to compile asthma‐related targets. Target data retrieved from each database were aggregated and deduplicated to establish a comprehensive pool of asthma‐associated targets. Subsequently, an overlap analysis was performed between the PCN‐predicted targets and those associated with asthma. The intersecting set of targets was subsequently illustrated by means of a Venn diagram. These intersecting targets were thereby identified as the putative therapeutic targets through which PCN may exert its effects against asthma.

#### 2.3.3 Construction of a Protein–Protein Interaction (PPI) Network for Immune‐Relevant Targets Derived From Active Components of PCN

The shared target genes were then submitted to the STRING database. Within this platform, a PPI network was generated under the following parameters: a minimum required confidence score of 0.9 (medium confidence) and removal of all disconnected nodes (i.e., those without any interactive links). The resulting network data were then exported and visualized using Cytoscape (version 3.8.0), after which the CytoNCA plugin was employed to compute key topological parameters for each node x′, including betweenness centrality (BC), degree centrality (DC), and closeness centrality (CC). Based on these parameters, the nodes were ranked in descending order according to their DC values, and those with the highest rankings were identified as putative core targets through which PCN may exert its therapeutic effects in the treatment of asthma. Core targets were identified through a two‐step filtering procedure: In the first round, nodes with DC, BC, and CC values all above the respective medians were retained; in the second round, the same median‐based filtering was applied to the subnetwork obtained from the first round. The nodes surviving both filtering steps were defined as putative core targets through which PCN may exert its therapeutic effects in the treatment of asthma.

#### 2.3.4 Gene Ontology (GO) and Kyoto Encyclopedia of Genes and Genomes (KEGG) Enrichment Analyses

To elucidate the functional implications of the core targets, GO and KEGG enrichment analyses were performed using the DAVID database, with a significance threshold set at *p* < 0.05. GO annotation encompassed three distinct functional categories: biological processes (BP), cellular components (CC), and molecular functions (MF). Concurrently, KEGG pathway enrichment analysis was conducted to identify the primary signaling pathways potentially modulated by these core targets. The resulting enrichment data were subsequently ranked based on *p*‐values, and the most significantly enriched terms were selected for graphical visualization to facilitate interpretation.

#### 2.3.5 Building a PCN–Compound–Target–Asthma Interaction Network

Following the identification of blood‐absorbed bioactive constituents of PCN by UHPLC‐Q‐Exactive HRMS, each compound's SMILES notation was fetched from the PubChem database. For those constituents that could not be retrieved from the database, their chemical structures were delineated using ChemDraw software, exported in MOL format, and subsequently converted to SMILES notations using the Open Babel toolkit. The obtained SMILES strings were then imported in batches into the SwissTargetPrediction database for target prediction. The resulting data were subsequently integrated and deduplicated to establish a comprehensive dataset of potential targets for PCN.

### 2.4 Molecular Docking

The SDF‐format 3D structures of the key bioactive constituents were obtained from the PubChem database (https://pubchem.ncbi.nlm.nih.gov/). A subsequent conversion to Protein Data Bank (PDB) format was performed using Open Babel. Meanwhile, the three‐dimensional conformations of the primary target proteins were fetched from the PDB repository. Preprocessing of both receptor proteins and ligand molecules was performed using AutoDock 4.2.6, which included hydrogenation, removal of water molecules, and definition of the active pocket. Semi‐flexible molecular docking calculations were then carried out using AutoDock Vina to obtain binding energy values, and the results were saved in PDBQT format before being converted to PDB format. Finally, visualization analysis was conducted using PyMOL (version 2.5.7), during which the receptor–ligand interaction sites, including the specific amino acid residues involved, were annotated. The three‐dimensional structure of the ADORA2A receptor was retrieved from the PDB (ID: 3PWH). This structure corresponds to the thermostabilized receptor in complex with the selective inverse agonist ZM241385 (inactive state conformation), as reported by Doré et al. (2011). 3PWH was selected because it provides a well‐defined ligand‐binding pocket and has been widely used as a reference structure in ADORA2A receptor docking studies. Despite a moderate resolution of 3.30 Å, the structure is well‐validated (*R*‐free = 0.318) and has been extensively applied in structure‐based virtual screening of ADORA2A receptor ligands.

### 2.5 Evaluating the Pharmacological Effects of PCN on Asthmatic Rats

#### 2.5.1 Experimental Animals and Study Groups

All animal procedures were carried out under approval from the Ethics Committee for Medical Experimental Animals of Anhui University of Chinese Medicine (Permit No. AHUCM‐rats‐2024108). This study used 56 male SD rats [Sprague–Dawley: mean weight 210 ± 30 g; certification SCXK (Yu) 2025‐0005], which we sourced from Skbex Biotechnology Co. Ltd. in Henan Province, China. The animals were maintained under controlled environmental conditions (temperature: 20 ± 2°C; humidity: 50 ± 15%) with free access to food and water and subjected to a 12‐h light/dark cycle for 1 week prior to the experiment.

Based on previously described methods (Ran et al. 2019; Ruan et al. 2023; Tao et al. 2022), the rats were randomly divided into seven groups (*n* = 8 per group): normal control (Control), model (Model), low‐dose PCN (PCN‐L, 3.645 g/kg), medium‐dose PCN (PCN‐M, 7.289 g/kg), high‐dose PCN (PCN‐H, 14.578 g/kg), positive control Dex (0.405 mg/kg), and Guilong Kechuanning (GLKCN, 0.405 g/kg). The PCN decoction and positive control drugs were administered by oral gavage once daily from Days 15 to 28. Except for the Control group, all rats were sensitized by intraperitoneal injection of 1 mL of 10% OVA solution, containing 100 mg OVA and 100 mg Al_2_(OH)₃, on experimental Days 1 and 8, following an established sensitization protocol from our previous studies. The Control group was treated with normal saline at an identical volume.

On experimental Day 15, the rats were placed in a custom‐made, partially enclosed nebulization chamber. Each experimental group was subjected to daily nebulized inhalation of 1% OVA solution to induce asthma, accompanied by cold stimulation, once daily for 30 min over 14 consecutive days. The control group was nebulized with normal saline under the same conditions.

#### 2.5.2 Body Mass and Bronchial Reactivity in a Rat Model

Across the oral treatment period, variations in the animals' physiological parameters were recorded. After following the final OVA challenge, juvenile rats in each group received a nebulized dose of 100 μL acetylcholine (Ach) solution, with concentrations increasing stepwise (3.125, 6.25, 12.5, 25, and 50 mg/mL). At each concentration level, the enhanced pause (Penh) and the instantaneous EF50 (expiratory flow at 50% vital capacity) were recorded continuously. These measurements served to evaluate the degree of airway responsiveness.

#### 2.5.3 H&E‐Based Pathological Staining

For histopathological evaluation, specimens of the lung, thymus, and spleen were fixed with 10% paraformaldehyde and then embedded in paraffin. Sections measuring 5–8 μm in thickness were prepared from the embedded tissues by means of an ultramicrotome. Subsequently, the prepared sections were examined under a light microscope to assess pathological and morphological alterations in the rat tissues.

#### 2.5.4 Determination of Serum Cytokines

The serum concentrations of tumor necrosis factor‐alpha (TNF‐α), immunoglobulin E (IgE), and cyclic adenosine monophosphate (cAMP) were determined by means of ELISA kits in compliance with the standard guidelines.

#### 2.5.5 Quantitative Real‐Time PCR (qRT‐PCR) Analysis

Total RNA was extracted from lung tissues using a TRIzol reagent (Invitrogen, Cat. No. 15596026) according to the manufacturer's instructions. RNA concentration and purity were determined using a NanoDrop 2000 spectrophotometer. Reverse transcription was performed using the PrimeScript RT Reagent Kit (Takara, Cat. No. RR037A) with 1 μg of total RNA per reaction. qPCR was carried out on a LightCycler 480 II system (Roche) using SYBR Premix Ex Taq (Takara, Cat. No. RR420A) under the following conditions: 95°C for 30 s, followed by 40 cycles of 95°C for 5 s and 60°C for 30 s. Each sample was run in triplicate. The 2^−^ΔΔCt method was used to quantify the relative expression of IL‐4, IL‐5, IL‐18, and IL‐1β transcripts. The primers (detailed in Table S1) were custom designed and purchased from Shanghai Shengong Biotechnology Co. Ltd.

#### 2.5.6 Western Blot Analysis

Protein extraction was performed by homogenizing 50 mg of lung tissue in pre‐cooled RIPA lysis buffer. After centrifugation, the supernatant was collected, and protein concentration was determined using the BCA method. Samples were mixed with loading buffer at a 4:1 ratio and heated at 100°C for 10 min to denature the proteins. Proteins were separated by SDS‐PAGE and transferred onto PVDF membranes using a wet transfer system. Membranes were blocked with 5% nonfat milk in TBST at room temperature for 2 h, followed by incubation with primary antibodies overnight at 4°C with gentle shaking. The following primary antibodies were used: rabbit anti‐ADORA2A (1:1000, Affinity, Cat. No. DF6401, Lot No. BA2406‐2), rabbit anti‐protein kinase A (PKA) gamma (1:1000, Yamay, Cat. No. R010375, Lot No. 529P02A21), rabbit anti‐phospho‐PKA (Thr197) (1:1000, Chengdu Zhengneng Biotechnology, Cat. No. 347334, Lot No. Q115F01), rabbit anti‐NLRP3 (1:1000, Abcam, Cat. No. ab263899, clone EPR23094‐1), and mouse anti‐β‐actin (1:5000, Proteintech, Cat. No. 66009‐1‐Ig) as the loading control. After three washes with TBST (10 min each), membranes were incubated with HRP‐conjugated secondary antibodies (goat anti‐rabbit IgG, 1:5000, Proteintech, Cat. No. SA00001‐2; goat anti‐mouse IgG, 1:5000, Proteintech, Cat. No. SA00001‐1) at room temperature for 2 h. Protein bands were visualized using an ECL substrate (Millipore, Cat. No. WBKLS0500) and captured using a Bio‐Rad ChemiDoc MP imaging system. Densitometric analysis was performed using ImageJ software, and relative protein expression levels were normalized to β‐actin.

### 2.6 Statistical Analysis

Quantitative results were expressed as mean ± SD. For endpoint measurements (including net weight gain, ELISA, qPCR, and western blot), one‐way ANOVA followed by LSD post hoc test was applied for multigroup comparisons. For longitudinal or dose–response measurements with repeated sampling from the same animals (including absolute body weight over time, Penh, and EF50 at escalating Ach concentrations), two‐way ANOVA with repeated measures followed by Tukey's post hoc test was used. Differences with *p* < 0.05 were deemed statistically significant.

## 3 Results

### 3.1 Elucidation of the Chemical Composition of PCN

UPLC‐Q‐Orbitrap MS with full MS/dd‐MS^2^ acquisition was employed to profile the chemical constituents of PCN. TICs for both ionization polarities are illustrated in Figure 1. Putative unknown substances were identified by aligning retention times, exact high‐resolution mass measurements, and MS/MS fragment ions, with the aid of TraceFinder software and comparative literature data. A total of 107 compounds were characterized in the present work. The identified classes included in Figure 2 as follows: 3 saccharides, 10 amino acids, 26 flavonoids, 2 alkaloids, 4 terpenoids, 6 coumarins, 1 lignan, 28 organic acids, 4 phenylpropanoids, 5 phenols, 5 nucleosides, and 14 other substances (such as aldehydes and aromatics). The detailed information on the identified compounds is provided in Table S2.

**FIGURE 1:**
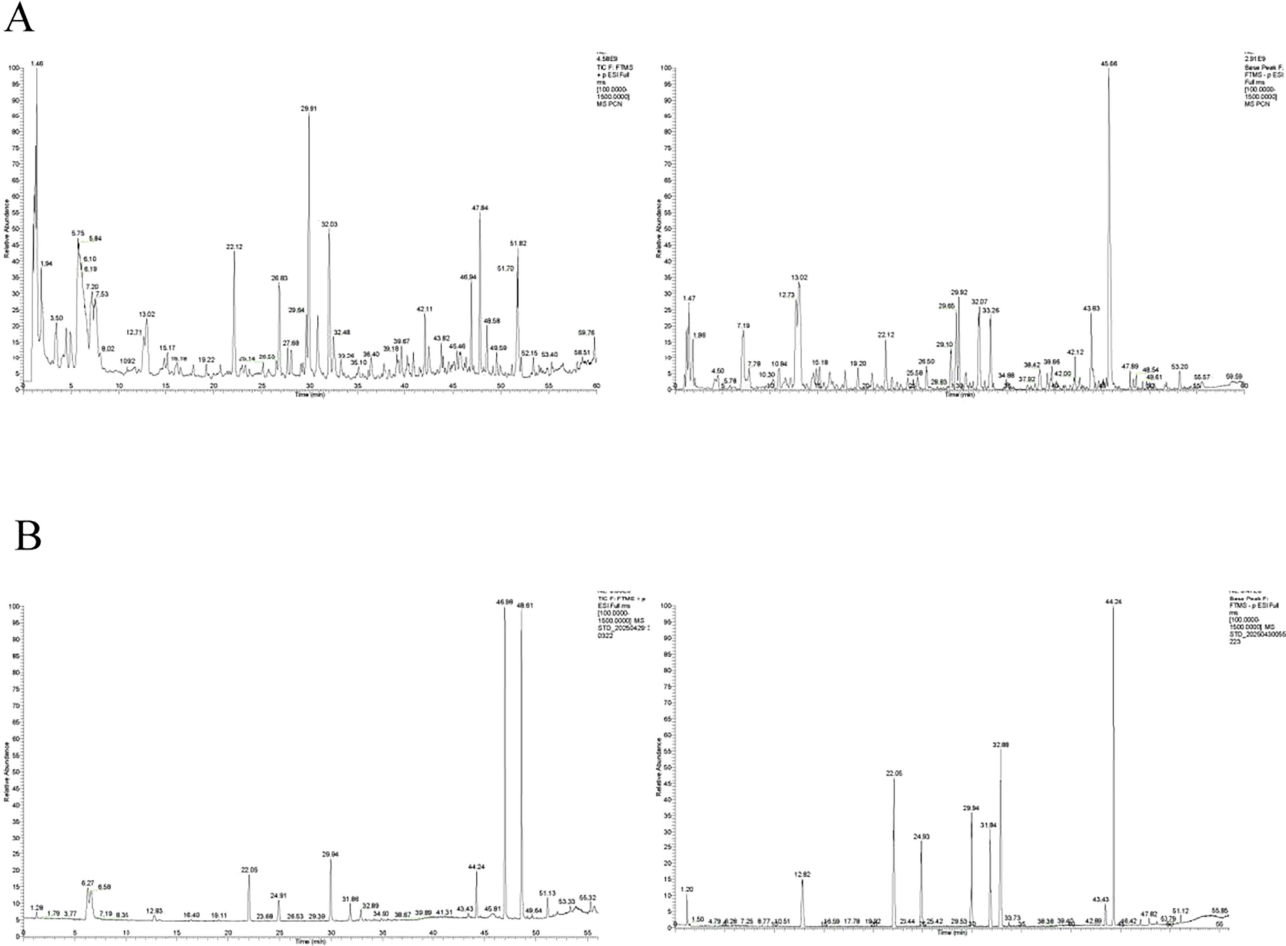
Total ion chromatogram (TIC) of Pingchuanning by UHPLC‐Q‐Orbitrap‐HRMS. (A) Ion chromatogram (TIC) of the standard by UHPLC‐Q‐Orbitrap‐HRMS(B). The left panel shows positive ion mode, and the right panel shows negative ion mode.

**FIGURE 2:**
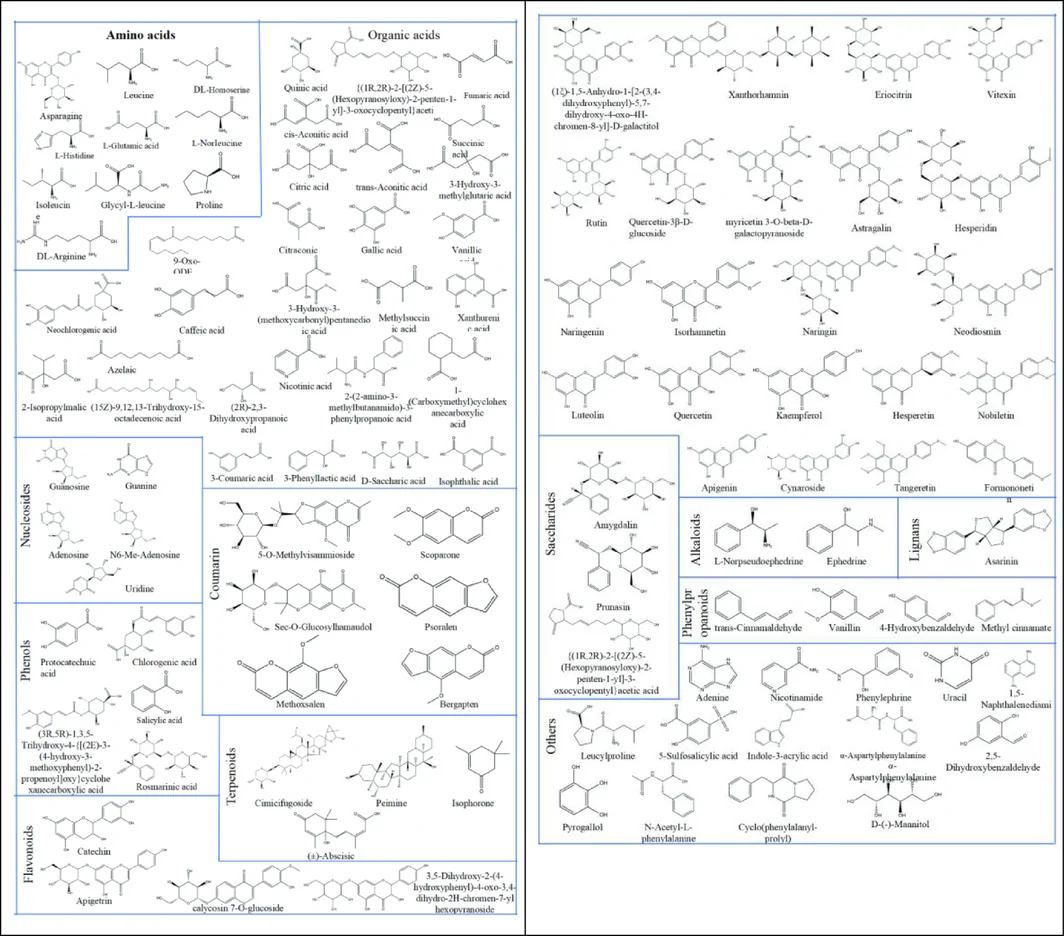
Structures of major compounds in PCN.

### 3.2 Identification of the Blood‐Absorbed Constituents of PCN

The chemical constituents of PCN absorbed into rat blood were analyzed using a UPLC‐Q‐Orbitrap mass spectrometer operating in full MS/dd‐MS^2^ scan mode. The TICs of PCN in both positive and negative ion modes are presented in Figure 3. Identification of the absorbed constituents was performed using the data processing software Xcalibur and Compound Discoverer, in conjunction with relevant literature, by comparing retention times, accurate masses, high‐resolution mass spectrometric data, and MS/MS fragmentation patterns. In this study, a total of 36 compounds were identified, comprising one saccharide, six amino acids, one flavonoid, two alkaloids, ten organic acids, two indoles, two coumarins, one phenylpropanoid, and nine other compounds (including phenols, aldehydes, and aromatics) in Table 1.

**FIGURE 3:**
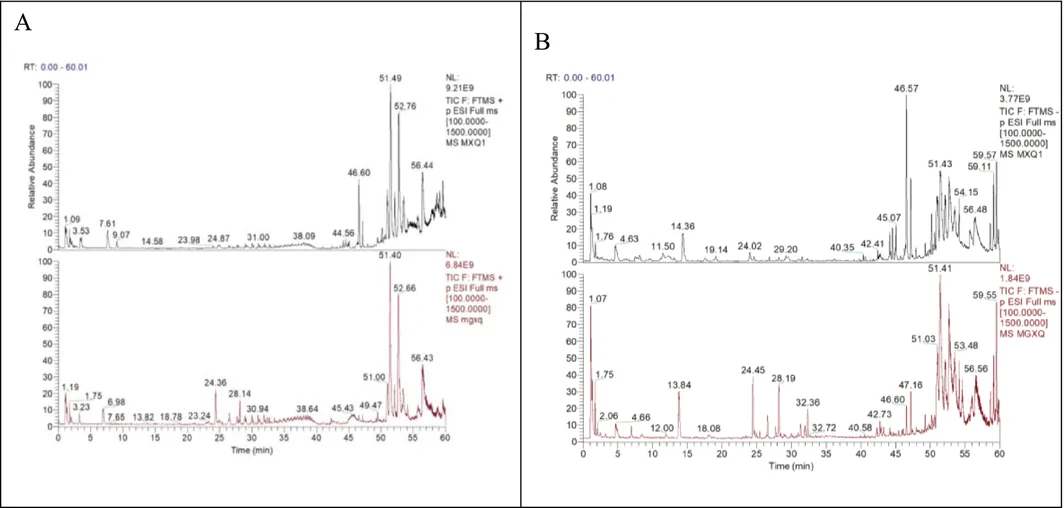
Total ion chromatograms of blank and PCN‐containing serum samples from normal and asthmatic model rats (A: positive ion modes; B: negative ion modes). From top to bottom: Blank serum (control) and PCN‐containing serum (medicated) were collected from asthmatic rats.

**TABLE 1:** Identification of blood‐absorbed components of Pingchuanning.

Name	RT (min)	Formula	Annot. ΔMass (ppm)	Reference ion	Classification	Observed m/z	MS2
L‐Histidine	1.02	C_6_H_9_N_3_O_2_	0.28	[M + H] + 1	Amino acids	155.06952	110.0712, 95.0604, 93.0447, 83.0603
DL‐Arginine	1.049	C_6_H_14_N_4_,O_2_	0.04	[M + H] + 1	Amino acids	174.11168	70.0651, 60.0556, 116.0706, 130,0.0975
L‐Glutamic acid	1.195	C_5_H_9_NO_4_	0.97	[M + H] + 1	Amino acids	148.0658	84.0444, 102.0550, 130.0500, 148.0605, 56.0495
Proline	1.216	C_5_H_9_NO_2_	0.78	[M + H] + 1	Amino acids	116.07069	70.0651, 98.0603
(2R)‐2,3‐Dihydroxypropanoic acid	1.259	C_3_H_6_O_4_	0.48	[M‐H]‐1	Organic acids	106.02666	75.0088, 72.9932, 59.0139
Nicotinamide	1.756	C_6_H_6_N_2_O	0.31	[M + H] + 1	Others	123.05533	78.0338, 80.0495
Uracil	1.757	C_4_H_4_N_2_O_2_	0.1	[M + H] + 1	Others	112.02728	96.0080, 70.0288
Citric acid	1.762	C_6_H_8_O_7_	0.38	[M‐H]‐1	Organic acids	191.0198	111.0089, 87.0088, 85.0296, 57.0347, 121.0195
Uridine	1.765	C_9_H_12_N_2_O_6_	−0.35	[M‐H]‐1	Nucleosides	243.06217	153.0308, 200.0565, 140.0355, 111.0201
4‐Hydroxybenzaldehyde	1.869	C_7_H_6_O_2_	0.49	[M + H] + 1	Phenylpropanoids	122.03684	95.0491, 77.0386, 67.0542
Succinic acid	2.061	C_4_H_6_O_4_	1.15	[M‐H]‐1	Organic acids	117.01947	117.0195, 99.0090, 73.0296
Leucylproline	2.063	C_11_H_20_N_2_O_3_	−0.41	[M + H] + 1	Others	228.1473	116.0704, 70.0651
Isoleucine	2.08	C_6_H_13_NO_2_	−0.18	[M + H] + 1	Amino acids	131.09461	86.0963, 69.0699
9‐Oxo‐ODE	45.18	C_18_H_30_O_3_	1.81	[M + H] + 1	Organic acids	294.22003	81.0701, 93.0700, 151.1123, 277.2165
Leucine	2.08	C_6_H1_3_NO_2_	−0.18	[M + H] + 1	Amino acids	131.09461	86.0963, 132.1019
3‐Hydroxy‐3‐methylglutaric acid	2.134	C_6_H_10_O_5_	0.46	[M‐H]‐1	Organic acids	162.0529	111.0089, 73.9932, 85.0296, 173.0093
3‐Hydroxy‐3‐(methoxycarbonyl)pentanedioic acid	2.716	C_7_H_10_O_7_	−0.18	[M‐H]‐1	Organic acids	206.04262	111.0089, 73.9932, 85.0296, 173.0093
Methylsuccinic acid	2.97	C_5_H_8_O_4_	0.69	[M‐H]‐1	Organic acids	132.04235	57.0347, 59.0139, 99.0463, 101.0245
Pyrogallol	3.153	C_6_H_6_O_3_	0.67	[M + H] + 1	Others	126.03178	109.0285, 99.0442, 81.0335
L‐Norpseudoephedrine	4.806	C_9_H_13_NO	0.23	[M + H] + 1	Alkaloids	152.10703	134.0966, 117.0700, 91.0545
Ephedrine	6.7	C_10_H_15_NO	0.43	[M + H] + 1	Alkaloids	166.12271	148.1122, 117.0700, 133.0887, 57.0336
1,5‐Naphthalenediamine	7.611	C_10_H_10_N_2_	0.38	[M + H] + 1	Others	158.08446	132.0809
Indole‐3‐acrylic acid	7.613	C_11_H_9_NO_2_	−0.33	[M + H] + 1	Indole	188.07054	146.0602, 144.0810, 118.0652, 115.0544
Sulfosalicylic acid	7.726	C_7_H_6_O_6_S	0.12	[M‐H]‐1	Others	217.98854	79,9576, 93.0347, 137.0245
2‐Isopropylmalic acid	9.369	C_7_H_12_O_5_	0.04	[M‐H]‐1	Organic acid	176.06848	85.0660, 115.0402, 129.0563
Amygdalin	12.863	C_20_H_27_NO_11_	1.44	[M‐H]‐1	Saccharides	456.15182	71.0140, 101.0246, 89.0246, 161.0455, 323.0984
Prunasin	16.315	C_14_H_17_NO_6_	0.63	[M‐H]‐1	Saccharides	294.09848	161.0458, 113.0248, 85.0297
3‐Phenyllactic acid	18.994	C_9_H_10_O_3_	0.78	[M‐H]‐1	Organic acids	165.05585	147,0454, 119.0505, 73.9933, 91.0555
*N*‐Acetyl‐L‐phenylalanine	21.09	C_11_H_13_NO_3_	0.86	[M‐H]‐1	Others	207.08972	164.0719, 147.0454, 91.0555, 58.0230
Salicylic acid	26.929	C_7_H_6_O_3_	1.7	[M‐H]‐1	Organic acids	137.02465	137.0244, 109.0295, 93.0348
2,5‐Dihydroxybenzaldehyde	26.938	C_7_H_6_O_3_	0.98	[M‐H]‐1	Others	138.03183	109.0298, 93.0348
5‐*O*‐Methylvisammioside	29.691	C_22_H_28_O_10_	1.31	[M + H] + 1	Coumarin	452.16884	291.1228, 273.1122, 243.0551
Azelaic acid	30.372	C_9_H_16_O_4_	1.53	[M‐H]‐1	Organic acids	187.09787	125.0974, 97.0661
*Sec*‐*O*‐glucosylhamaudol	38.909	C_21_H_26_O_10_	1.5	[M + H] + 1	Coumarin	439.16053	277.1073, 259.0966, 205.0497
(15Z)‐9,12,13‐Trihydroxy‐15‐octadecenoic acid	43.665	C_18_H_34_O_5_	1.31	[M‐H]‐1	Organic acid	330.24106	171.1030, 127.1129, 199.1343
Nobiletin	46.769	C_21_H_22_O_8_	1.81	[M + H] + 1	Flavonoids	403.13943	373.0924, 229.0347, 165.0553

### 3.3 Results of the Construction of Network Pharmacology

#### 3.3.1 Construction of Disease Targets

Using the SwissTargetPrediction database, 507 potential targets for PCN were identified after prediction and deduplication. Asthma‐related targets were retrieved from the GeneCards, DisGeNET, DrugBank, PharmGKB, OMIM, and TTD databases, yielding 9321 targets after integration and deduplication. As shown in Figure 4A, Venn analysis of the two target sets revealed 349 overlapping targets, which were considered potential targets underlying the anti‐asthmatic effects of PCN.

**FIGURE 4:**
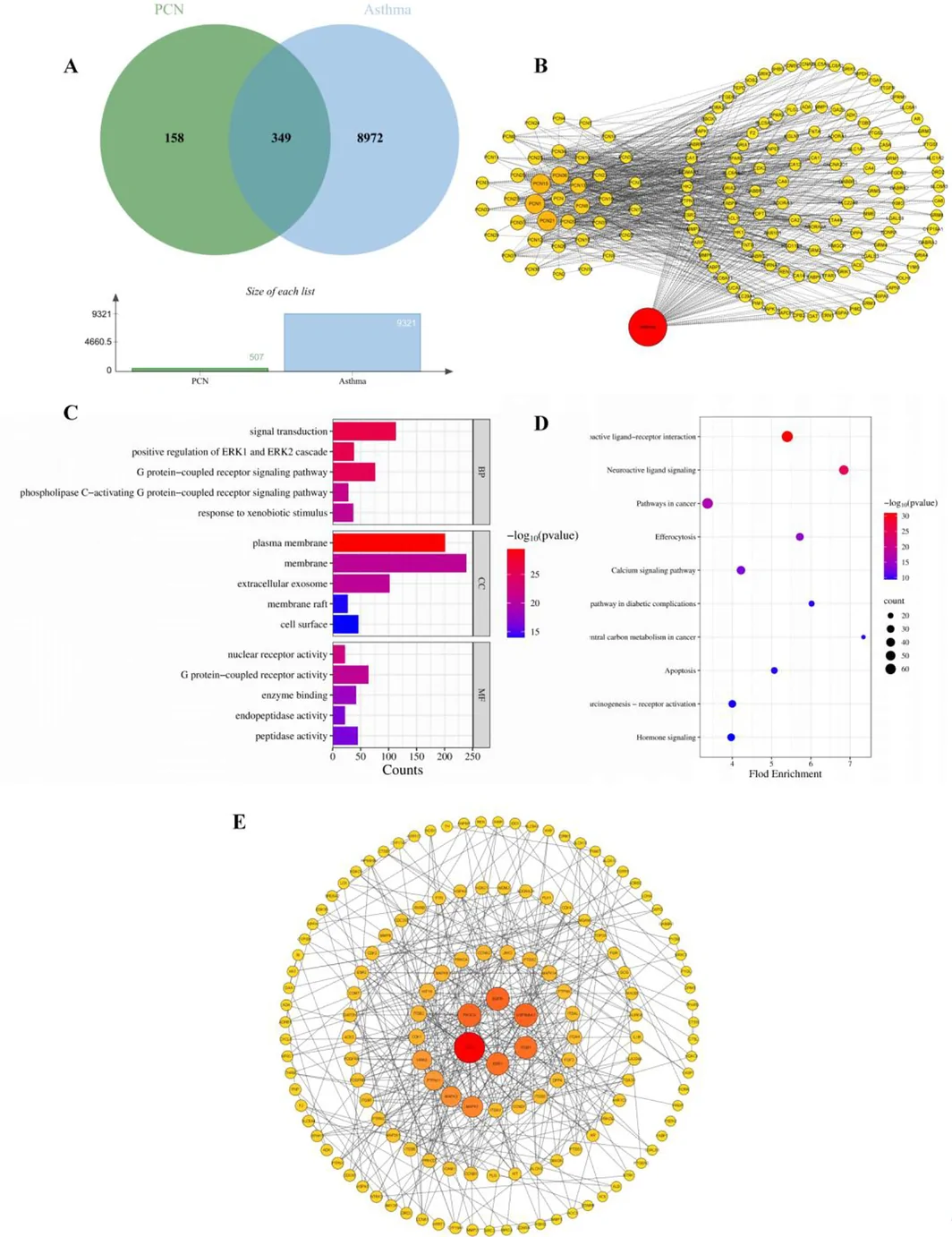
Network pharmacological analysis of PCN for the treatment of asthma. (A) Venn diagram of targeting PCN against asthma. (B) Component‐target interaction network. (C) Secondary classification analysis of KEGG pathway of PCN. (D) GO analysis. (E) PCN intersection target PPI with immune diseases.

#### 3.3.2 Construction of PPI Network

A PPI network consisting of 349 nodes was constructed based on the STRING database. Through visualization using Cytoscape 3.8.0 and topology analysis using the CytoNCA plugin, DC values were used as the mapping basis for node size and color. The larger the node size and the darker the color, which is illustrated in Figure 4B, the higher the DC value representing the target and the stronger its importance in the network.

#### 3.3.3 Results of GO and KEGG Enrichment Analyses

DAVID enrichment analysis yielded 835 GO terms, comprising 883 BP terms, 138 CC, terms and 254 MF terms. The top five terms from each category were selected for bar chart visualization, which is illustrated in Figure 4C. KEGG pathway enrichment analysis identified 181 relevant pathways, from which the ten most significantly enriched pathways were selected for bubble plot representation. In the bubble plot, the gene ratio reflects the proportion of pathway targets relative to the total core targets, the bubble size corresponds to the count value (the number of enriched targets), and the color intensity represents the significance level of the *p*‐value, with deeper red indicating higher confidence. As illustrated in Figure 4D, the results showed that the potential therapeutic mechanisms of PCN active ingredient targets in asthma are primarily involved in pathways such as the neuroactive ligand–receptor interaction signaling pathway, pathways in cancer, and the calcium signaling pathway.

#### 3.3.4 Construction of the “PCN–Target–Disease” Network

The “PCN–target–disease” network was constructed using Cytoscape (version 3.8.0). Following DC filtering, the final network comprised 37 active ingredient nodes and 116 core target nodes. The network was visualized based on DC values calculated by the CytoNCA plugin, with nodes represented in a size‐ and color‐gradient manner—larger and darker nodes indicate higher DC values of the corresponding target proteins. Edges represent the interactions between PCN and core targets, as well as between asthma and core targets. As shown in Figure 4E, the results indicate that the ten gene targets closely related to PCN and asthma are ADORA2A, ADORA3, carbonic anhydrase (CA)1, CA2, CA9, CA12, CACNA2D, GABBR, SLC22A6, LTA4H.

#### 3.3.5 Molecular Docking Validation

Based on the constructed “PCN–target–disease” network, multiple absorbed active components of PCN were found to exhibit potential targeting interactions with ADORA2A, a G protein‐coupled receptor (GPCR) involved in the calcium signaling pathway. Using the SwissTargetPrediction database, seven active components were identified as having potential interactions with ADORA2A: 5‐*O*‐methylvisammioside, 9‐oxo‐ODE, nobiletin, *sec*‐*O*‐glucosylhamaudol, amygdalin, uridine, and prunasin. To validate the binding activity of PCN‐derived active compounds with asthma‐related targets, ADORA2A was selected as a core target for molecular docking with these seven components. Among them, 5‐*O*‐methylvisammioside, 9‐oxo‐ODE, nobiletin, and *sec*‐*O*‐glucosylhamaudol exhibited moderate binding affinities to the target, suggesting their potential as key bioactive constituents, as shown in Figure 5. Visualization using PyMOL revealed that nobiletin formed three binding sites; ADORA2A, 9‐Oxo‐ODE, and *sec*‐*O*‐glucosylhamaudol each formed two sites; and 5‐*O*‐methylvisammioside formed four sites. The respective binding energies were −4.67, −4.17, −3.96, and −3.82 kcal/mol, indicating that these compounds may contribute to the pathological modulation of asthma through regulation of ADORA2A and related targets. To validate the docking protocol, the known ADORA2A agonist CGS21680 was docked into the same receptor structure as a positive control {Lebon, 2015 #171}. The best docking pose of CGS21680 exhibited a binding energy of −5.48 kcal/mol and formed three hydrogen‐bonding interactions with residues in the binding pocket, confirming that the docking procedure correctly identified the known binding site of this ADORA2A receptor ligand.

**FIGURE 5:**
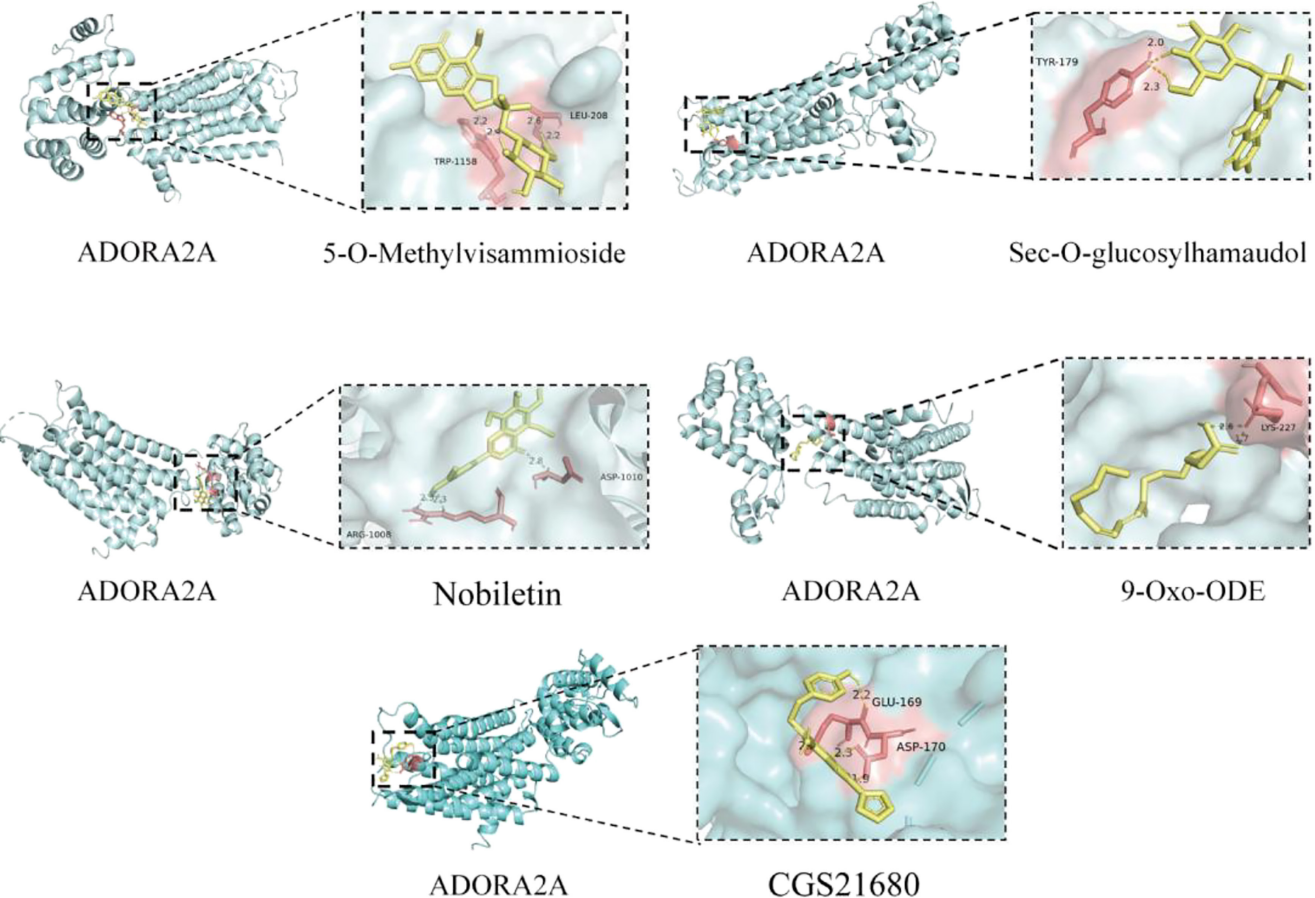
Molecular docking results of four PCN‐derived active components with ADORA2A.

### 3.4 Ameliorative Effects of PCN on Asthmatic Rats

#### 3.4.1 Effects of PCN on Body Weight and Airway Responsiveness

Following OVA challenge, the rats exhibited pronounced respiratory symptoms during routine feeding, including tachypnea, wheezing, abdominal contractions, and audible wheezing sounds. The dynamic changes in absolute body weight over the experimental period are shown in the right panel of Figure 6A: The body weight of rats in the control group exhibited a consistent and steady increase throughout the experimental period, whereas the model group displayed a significantly slower growth trend. Following intervention with different doses of PCN, a notable improvement in body weight recovery was observed, indicating that PCN exerted a stabilizing effect on weight gain. Furthermore, the left panel of Figure 6A presents the net weight gain (ΔWeight = final weight − initial weight) of each group during the experimental period. Consistent with the absolute weight trend, PCN treatment dose‐dependently increased net weight gain compared with the model group, further supporting the beneficial effect of PCN on body weight recovery.

**FIGURE 6:**
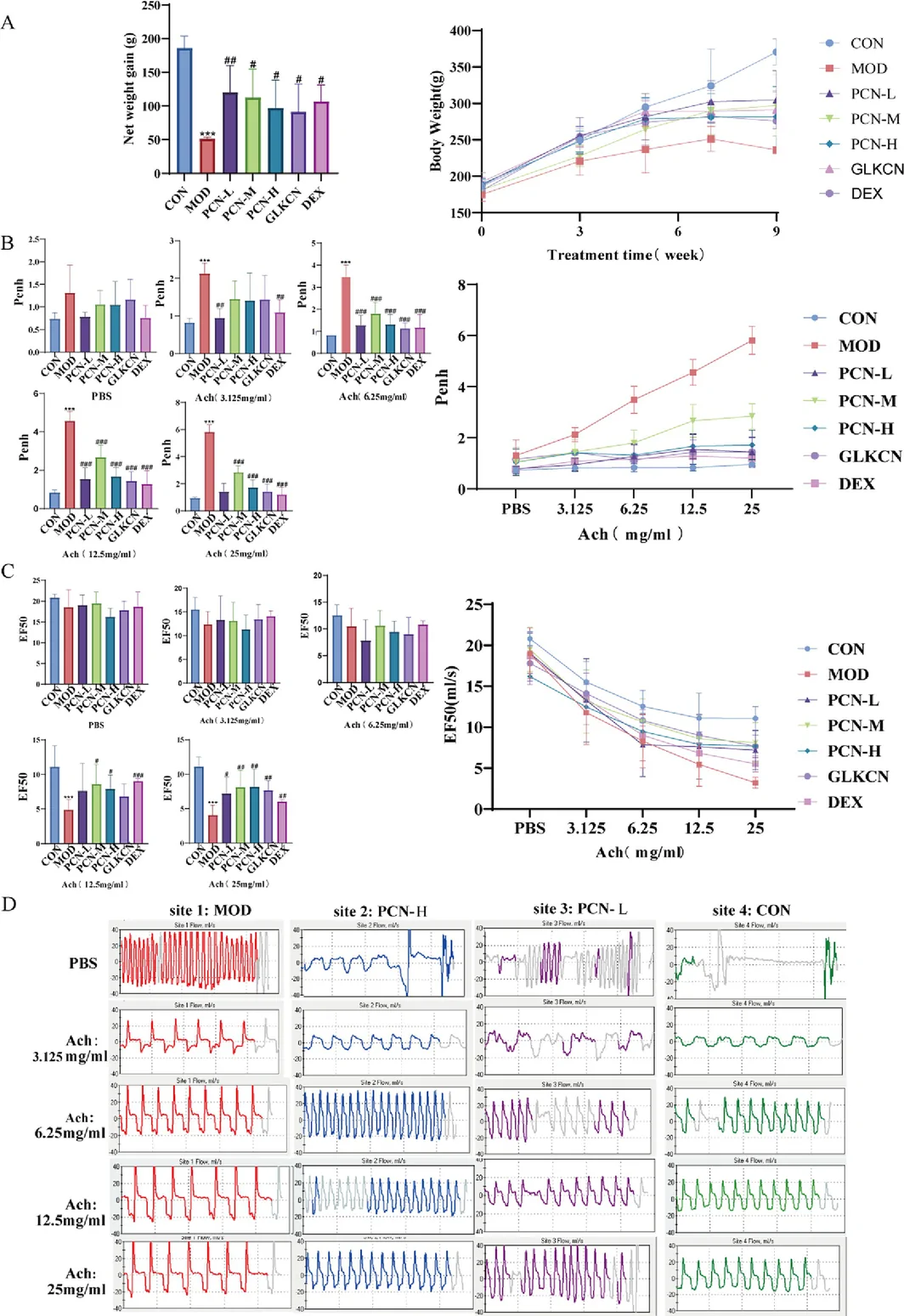
Effects of PCN on body weight and airway responsiveness. (A) Body weight changes. Left panel: net weight gain (ΔWeight = final weight − initial weight) of rats in each group during the experimental period. Data are presented as mean ± SEM, *p < 0.05, **p < 0.01, ***p < 0.001 vs. CON group; #*p* < 0.05, ##*p* < 0.01, ###*p* < 0.001 vs. MOD group (one‐way ANOVA followed by LSD post hoc test). Right panel: dynamic changes in absolute body weight over the experimental period (weeks).(two‐way ANOVA with repeated measures followed by Tukey's post hoc test). (B) Enhanced pause (Penh) in response to increasing concentrations of acetylcholine (Ach). (C) 50% expiratory flow (EF50) in response to increasing concentrations of Ach. (D) Representative flow waveforms. Each row represents one Ach concentration (PBS, 3.125, 6.25, 12.5, 25 mg/mL); each column represents a group (MOD, PCN‐H, PCN‐L, CON). Flow signals were sampled at 0.1 Hz (one data point every 10 s) and connected by lines for clarity. The *x*‐axis is time (s), and the *y*‐axis is flow rate (mL/s); all waveforms are displayed with the same scale to allow direct comparison. Data are presented as mean ± SEM, *p < 0.05, **p < 0.01, ***p < 0.001 vs. CON group; #*p* < 0.05, ##*p* < 0.01, ###*p* < 0.001 vs. MOD group (two‐way ANOVA with repeated measures followed by Tukey's post hoc test).

Upon nebulization with methacholine at concentrations of 3.125, 6.25, 12.5, and 25 mg/mL, as illustrated in Figure 6B, the Penh values in the model group were significantly elevated compared with those in the control group (*p* < 0.01). Furthermore, at methacholine concentrations of 6.25, 12.5, and 50 mg/mL, all PCN‐treated groups displayed markedly reduced Penh values relative to the model group (*p* < 0.01), indicating that PCN attenuated airway hyperresponsiveness in asthmatic rats to varying degrees. The EF50 values (Figure 6C) reflected impaired small airway function. In contrast, all treatment groups and positive controls markedly alleviated the reduction in EF50 (*p* < 0.001) in 12.5 and 25 mg/mL, suggesting a protective effect against small airway dysfunction.

To visually capture the morphological characteristics of airway obstruction, representative respiratory flow waveforms were recorded under increasing concentrations of Ach (Figure 6D). In this figure, each column represents a different group (CON, MOD, PCN‐H, PCN‐L), and each row represents the same Ach concentration (the first row corresponds to PBS baseline, followed by 3.125, 6.25, 12.5, and 25 mg/mL). The sampling frequency was 0.1 Hz (one data point every 10 s). The results showed that in the MOD group, the waveforms were generally symmetrical under PBS baseline. With increasing Ach concentration, the peak expiratory flow markedly decreased, and a pronounced expiratory plateau appeared during the late expiratory phase, indicating severe airway obstruction; respiratory rhythm disturbances were also observed at the highest concentration. In contrast, the PCN‐H and PCN‐L groups exhibited substantially attenuated waveform distortion and improved expiratory prolongation, with the PCN‐H group showing more pronounced effects. The normal control group (CON) maintained regular waveforms across all concentrations, with only a mild expiratory prolongation observed at the highest concentration.

Collectively, these findings demonstrated that asthmatic model rats exhibited dose‐dependent airway hyperresponsiveness to Ach challenge, characterized by elevated peak expiratory flow, reduced EF50, and abnormal expiratory waveforms. PCN treatment ameliorated these asthmatic manifestations, indicating that PCN exerted a significant anti‐airway hyperresponsiveness effect.

#### 3.4.2 PCN Ameliorated Lung Histopathological Injury and Decreased Inflammatory Cytokine Levels

The lung tissue is composed of the conducting portion and the respiratory portion. The conducting portion primarily features bronchioles with relatively prominent smooth muscle in their walls, while the respiratory portion mainly consists of alveoli, where the alveolar septa are rich in capillary networks—structures that collectively enable gas exchange. As shown in Figure 7A, in the control group, the alveolar and pulmonary tissue structures remained intact, with no significant inflammatory cell infiltration observed around the bronchi and blood vessels. In contrast, the model group exhibited severe pulmonary tissue damage, characterized by alveolar atrophy, collapse, and fusion, along with marked thickening of the alveolar walls. The bronchial walls showed smooth muscle hypertrophy, resulting in pronounced luminal narrowing, shedding of epithelial cells, and obvious inflammatory cell infiltration in the surrounding areas. Compared with the model group, the PCN‐treated groups demonstrated varying degrees of improvement in both pulmonary tissue destruction and inflammatory cell infiltration.

**FIGURE 7:**
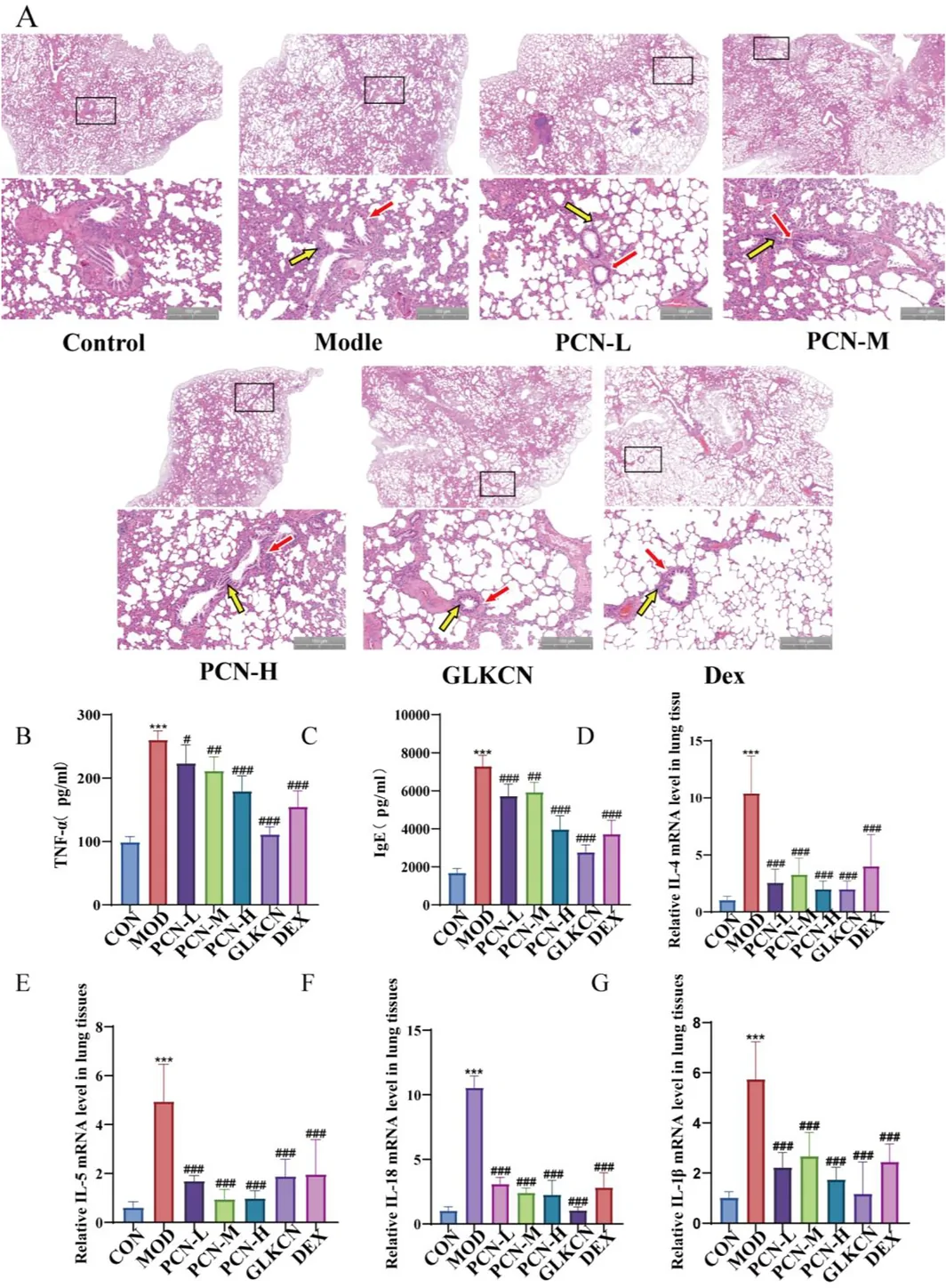
PCN affects cytokine secretion in lung tissues and BALF of asthmatic rats. (A) Results of H&E staining. (B) The concentration of TNF‐α in BALF. (C) The concentration of IgE in BALF. (D) Relative IL‐4 mRNA level in lung tissues. (E) Relative IL‐5 mRNA level in lung tissues. (F) Relative IL‐18 mRNA level in lung tissues. (G) Relative IL‐1β mRNA level in lung tissues. The mean ± SD is employed to express the results, *n* = 6, *p < 0.05; **p < 0.01, ***p < 0.001 vs. the CON, #*p* < 0.05, ##*p* < 0.01, ###*p* < 0.001 vs. the MOD.

#### 3.4.3 PCN Affected Cytokine Secretion in Lung Tissues and BALF of Asthmatic Rats

As shown in Figure 7B–G, the levels of TNF‐α, IL‐1β, and IL‐18 and the IL‐1β/IL‐18 ratio in lung tissues were significantly increased in the model group compared with the control group (*p* < 0.01, *p* < 0.001). Treatment with PCN‐L, PCN‐H, GLKCN, and Dex markedly reduced the elevated levels of these cytokines and the ratio compared to the model group (*p* < 0.01 or *p* < 0.001). Notably, PCN‐H and GLKCN exhibited more pronounced inhibitory effects on TNF‐α and IL‐1β production.

#### 3.4.4 Western Blot Analysis

As shown in the results (Figure 8A–E), compared with the normal control group (CON), the model group (MOD) exhibited significantly decreased levels of ADORA2A protein expression, intracellular cAMP content, and PKA phosphorylation level (p‐PKA/PKA ratio), while the protein expression of NLRP3 was remarkably upregulated. After intervention with different doses of PCN, compared with the MOD group, the PCN treatment groups dose‐dependently upregulated ADORA2A expression, elevated intracellular cAMP levels, and increased the p‐PKA/PKA phosphorylation ratio. Meanwhile, PCN administration notably suppressed the excessive overexpression of the NLRP3 inflammatory sensor. Collectively, these findings indicate that PCN could effectively activate the ADORA2A/cAMP/PKA signaling pathway and inhibit the overactivated NLRP3‐mediated inflammatory cascade, thereby exerting protective and anti‐inflammatory effects in asthma.

**FIGURE 8:**
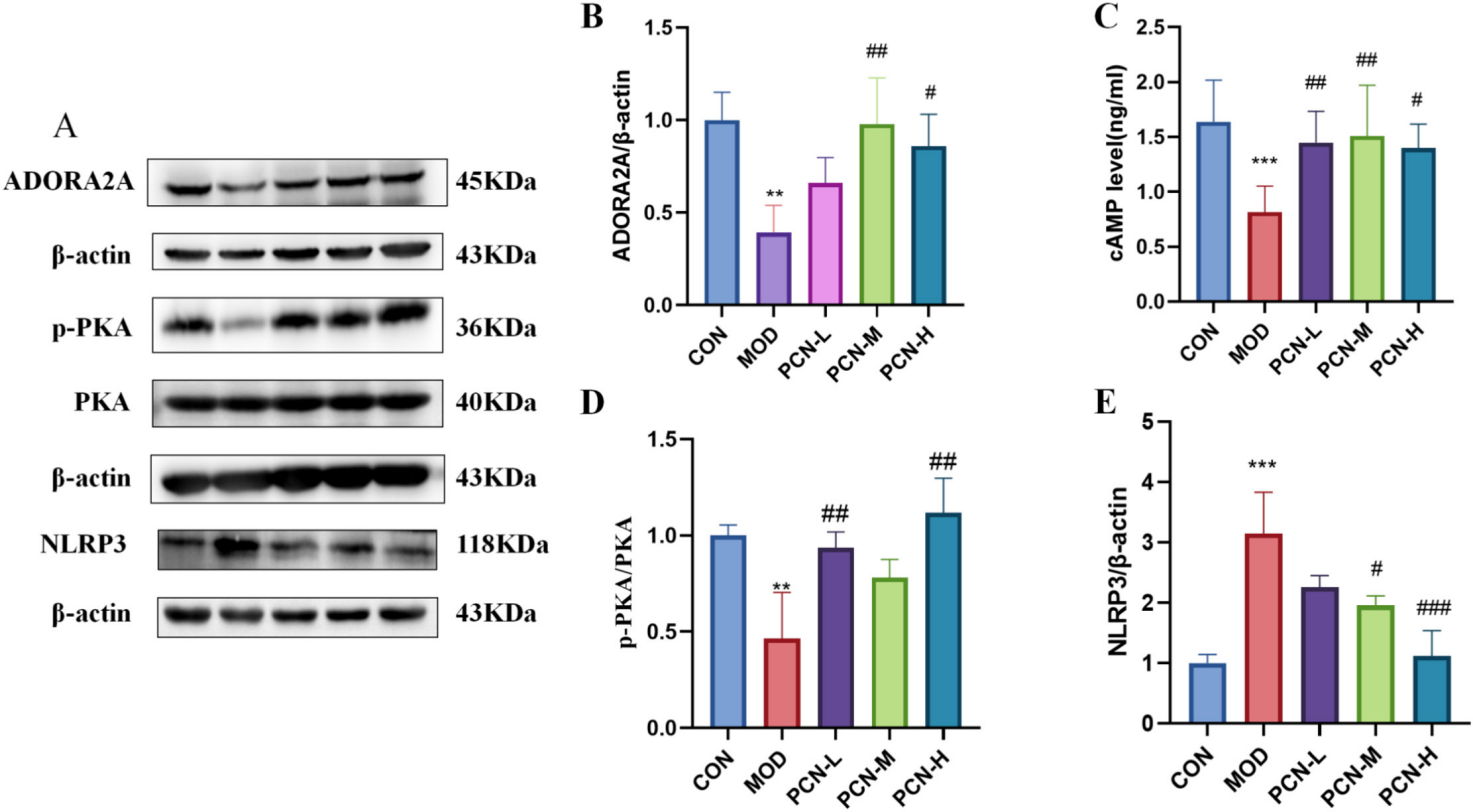
Western blot analysis. (A–E) Western blot analysis of ADORA2A, p‐PKA, PKA, and NLRP3 protein expression. The mean ± SD is employed to express the results, *n* = 6. ^#^ *p* < 0.05, ^##^ *p* < 0.01, ^###^ *p* < 0.001 vs. the MOD, *p < 0.05; **p < 0.01, ***p < 0.001 vs. the CON.

## 4 Discussion

Bronchial asthma is a globally prevalent chronic inflammatory airway disease characterized by airway hyperresponsiveness, persistent inflammation, and reversible airflow limitation (Agache et al. 2021; Shipp et al. 2023), imposing substantial burdens on patients and healthcare systems. Although inhaled corticosteroids and β_2_‐adrenoceptor agonists remain the first‐line therapeutic options, long‐term use is associated with drug dependence, adverse effects, and insufficient intervention in airway remodeling, underscoring the urgent need for safer and more effective treatments (Abu‐Helo et al. 2025; Comer et al. 2015; von Bülow et al. 2025). TCM offers unique advantages in asthma management through holistic regulation and multi‐target intervention. PCN, an empirical formula derived from the classic prescriptions Shegan Mahuang Decoction and Sanzi Yangqin Decoction, is clinically employed to warm the lung, resolve phlegm, relieve cough, and suppress wheezing. Despite its well‐documented efficacy, the pharmacodynamic material basis and precise molecular mechanisms of PCN in asthma treatment remain incompletely understood. The present study integrated UHPLC‐Q‐Orbitrap HRMS, network pharmacology, molecular docking, and in vivo experimental validation to systematically investigate the active components and mechanisms of PCN in mitigating asthma. The results confirmed that PCN exerts anti‐asthmatic effects primarily through the ADORA2A–PKA–cAMP–NLRP3 signaling axis, providing a scientific basis for its clinical application and mechanistic exploration.

PCN is formulated based on the classical TCM prescriptions Shegan Mahuang Decoction and Sanzi Yangqin Decoction, primarily composed of exterior‐releasing herbs combined with cough‐ and asthma‐relieving agents, supplemented with deficiency‐tonifying herbs, aiming to warm the lung, resolve phlegm, descend rebellious qi, relieve cough and asthma, and tonify deficiency, thereby addressing both symptoms and root causes. Among its constituents, Ephedrae herba (Mahuang) serves as the principal herb for dispersing lung pathogens. Modern studies have demonstrated that its bioactive components, ephedrine and pseudoephedrine, exhibit sympathomimetic effects by relaxing bronchial smooth muscle and alleviating asthma (Hu et al. 2024; Ren et al. 2025) and are widely used in Kampo medicine and European phytotherapy for symptomatic treatment of colds, bronchial asthma, and nasal congestion (Gad et al. 2021). The active constituent of Armeniacae semen amarum (Kuxingren), amygdalin, is decomposed in vivo to produce trace amounts of hydrogen cyanide, which inhibits the respiratory center to exert antitussive and anti‐asthmatic effects (Wei et al. 2023; Xing et al. 2026; A. N. Zhang, Li, et al. 2023). When combined with Ephedrae herba (as in the classic formula San'ao Decoction), it exhibits synergistic efficacy and reduced toxicity (Ma et al. 2021). Citri reticulatae pericarpium (Chenpi) has been shown to possess expectorant, anti‐inflammatory, and antioxidant properties, largely attributed to its flavonoid components such as hesperidin (Lee et al. 2023; P. Yu et al. 2024; X. Yu et al. 2018). Pinelliae rhizoma (Banxia), a key herb for resolving phlegm, is used after processing to reduce toxicity. Pharmacological studies indicated that its alkaloids and polysaccharides exert antitussive, expectorant, and anti‐emetic effects, and it is a frequently used herb in classic formulas such as Xiaoqinglong Decoction and Erchen Decoction (R. Wang, Xiao, et al. 2025; Z. Wang, Lu, et al. 2025).

A considerable proportion of the absorbed serum components consisted of amino acids, organic acids, and nucleosides, all of which are also endogenously present in the host organism. Although these compounds naturally occur in rat serum, their presence in the PCN decoction was confirmed by matching retention times and tandem mass spectrometry spectra, thereby verifying their exogenous contribution. The inclusion of these components in the absorbed compound inventory may reflect three possible scenarios: direct absorption of small‐molecule constituents from PCN, modulation of host metabolism by PCN, or a combination of both. To address this complexity, these compounds were individually annotated and categorized in Table 1, and the network pharmacology analysis was focused on compounds with higher structural specificity and documented pharmacological activity. Nevertheless, it should be noted that the potential overinclusion of endogenous metabolites may introduce bias in target prediction, and subsequent investigations should employ quantitative metabolomic approaches to differentiate exogenous absorption from metabolic responses.

These findings suggested that ADORA2A is a key target mediating the anti‐asthmatic effects of PCN, and the aforementioned four coumarin and flavonoid compounds represented the core active ingredients. The low‐dose PCN group (PCN‐L) showed limited efficacy in certain inflammatory parameters, such as TNF‐α (Figure 7B), where the reduction did not reach statistical significance versus the model group. In contrast, PCN‐M and PCN‐H produced significant, dose‐dependent reductions in TNF‐α, IL‐1β, and IL‐18, consistent with a pharmacological gradient. This may reflect a threshold effect, as the low dose (3.645 g/kg, roughly half the clinical equivalent) likely falls below the effective threshold for cytokines with lower sensitivity to modulation. Additionally, the complex, multi‐pathway regulation of TNF‐α may render it less responsive to low‐dose intervention than NLRP3 downstream effectors like IL‐1β and IL‐18. Importantly, the observed dose–response gradient (PCN‐H > PCN‐M > PCN‐L) strengthens the credibility of our findings and supports the need for adequate dosing in clinical practice to ensure optimal anti‐inflammatory efficacy.

UHPLC‐Q‐Orbitrap HRMS was employed to analyze the chemical composition of PCN and its absorbed components in the bloodstream. A total of 107 compounds were identified in PCN decoction, encompassing flavonoids, coumarins, alkaloids, organic acids, and amino acids. Among these, 36 constituents were absorbed into the serum of asthmatic rats and were therefore considered as potential directly acting bioactive components, including small‐molecule compounds such as 5‐*O*‐methylvisammioside, nobiletin, *sec*‐*O*‐glucosylhamaudol, ephedrine, and amygdalin. These absorbed components can enter the circulatory system and interact with disease‐related targets. Network pharmacology enables the systematic construction of a “component–target–pathway” network, thereby revealing the holistic mechanism by which multiple constituents in a TCM formula synergistically exert therapeutic effects (Greene and Loscalzo 2017; Zhang, Wang, and Li 2023). To predict the core targets and pathways of PCN in asthma treatment, 507 predicted targets of PCN components were intersected with 9321 asthma‐related targets, yielding 349 common targets. PPI network and topological analysis identified ADORA2A as the most critical core target, which was closely associated with calcium signaling and inflammatory regulation. KEGG enrichment analysis further demonstrated that the targets were predominantly enriched in pathways such as neuroactive ligand–receptor interaction and calcium signaling, both of which remained involved in airway smooth muscle contraction and immune‐inflammatory responses. Molecular docking results showed that 5‐*O*‐methylvisammioside, nobiletin, *sec*‐*O*‐glucosylhamaudol, and 9‐oxo‐ODE exhibited moderate binding affinities (binding energies ranging from −3.82 to −4.67 kcal/mol) to ADORA2A, with stable binding energies and multiple interaction sites. These findings suggested that ADORA2A is a key target mediating the anti‐asthmatic effects of PCN, and the aforementioned four coumarin and flavonoid compounds represented the core active ingredients.

To further validate the pharmacodynamics and mechanism of action of PCN, an asthmatic rat model was established using OVA combined with cold stimulation for in vivo experiments. Relevant studies have demonstrated that OVA combined with cold stimulation mimics the typical pathogenesis of human allergic asthma, induces Type I hypersensitivity (Matsuoka et al. 2000), and generates a stable and reliable animal model, thereby faithfully recapitulating the key pathological features of asthma, including immune imbalance, chronic airway inflammation, airway hyperresponsiveness, and lung histopathological damage. The proliferation and differentiation of T cells play a central driving role in this pathological process. Under asthmatic conditions, immune homeostasis is profoundly disrupted, and core immune organs such as the thymus and spleen exhibit structural damage and functional decline, failing to provide a normal microenvironment for the development and maturation of immune cells. Both adaptive and innate immunity are abnormally activated, ultimately resulting in an immune imbalance characterized by Th2‐skewed polarization, excessive release of pro‐inflammatory cytokines, and hyperactivation of the inflammasome (Kato 2019). In this process, aberrant T‐cell proliferation and pathological differentiation are key events that initiate and sustain chronic airway inflammation. Persistent antigen stimulation drives excessive T‐cell proliferation and an abnormal shift of CD4^+^ T cells toward the Th2 phenotype, disrupting the Th1/Th2 balance (Russkamp et al. 2019). This leads to substantial secretion of Type 2 inflammatory cytokines such as IL‐4 and IL‐5, promotes B cell production of IgE, recruits eosinophil infiltration into the airways, and further activates the NLRP3 inflammasome, promoting the maturation and release of pro‐inflammatory cytokines including IL‐1β and IL‐18 (Busse et al. 2021; Girodet et al. 2013; Hur et al. 2021; Robinson et al. 2017; Yoshimoto et al. 2009). These events established a vicious cycle of T‐cell dysfunction, inflammatory cascade amplification, airway hyperresponsiveness, and tissue injury, ultimately culminating in typical asthmatic manifestations such as wheezing, cough, and airway remodeling. The results of the present study demonstrated that PCN effectively reversed asthma‐induced immune aberrations. PCN not only repaired damaged immune organ structures, thereby providing a stable internal environment for normal T‐cell proliferation and differentiation but also suppressed aberrant T‐cell proliferation and pathological polarization, downregulated the expression of inflammatory cytokines such as IL‐4 and IL‐5, and corrected the Th1/Th2 immune imbalance. Moreover, by upregulating ADORA2A expression and subsequently elevating cAMP levels and enhancing PKA phosphorylation, PCN activated an immunoregulatory signaling axis that inhibited excessive NLRP3 inflammasome activation and blocked the inflammatory cascade. Consequently, PCN ameliorated immune dysfunction, alleviated lung tissue injury and airway inflammation, and ultimately achieved therapeutic effects against asthma.

Western blot analysis further elucidated the molecular mechanism by which PCN regulated inflammation. Compared with the model group, PCN treatment significantly upregulated ADORA2A expression {Wang, 2019 #166}, elevated cAMP content, and enhanced PKA phosphorylation (as reflected by the increased p‐PKA/PKA ratio), while downregulating NLRP3 inflammasome protein expression. ADORA2A is an important GPCR that, upon ligand binding, activates adenylyl cyclase via the stimulatory G protein (Gs), leading to an increase in intracellular cAMP production. Elevated cAMP subsequently activates PKA through the binding of cAMP to its regulatory subunits, which triggers the release and activation of the catalytic subunits (as reflected by the increased p‐PKA/PKA ratio). Activated PKA then phosphorylates downstream effectors that converge on the suppression of NLRP3 inflammasome activation, thereby reducing the release of pro‐inflammatory cytokines such as IL‐1β and IL‐18 {Zhang, 2026 #167} {Chi, 2025 #168}. This ADORA2A → cAMP → PKA → NLRP3 signaling axis thus represents a key anti‐inflammatory mechanism through which PCN exerts its therapeutic effects in asthma. The NLRP3 inflammasome played a critical role in asthma pathogenesis by promoting caspase‐1 activation and the secretion of IL‐1β and IL‐18, which exacerbated airway inflammation and hyperresponsiveness. These findings indicated that activation of the ADORA2A‐PKA‐cAMP signaling axis and subsequent inhibition of NLRP3‐mediated inflammatory cascades constituted the core mechanism underlying the anti‐asthmatic effects of PCN.

Accumulating evidences suggested that downstream of cAMP signaling, there exists a complex regulatory network beyond the classical PKA pathway. Ca^2+^ signaling plays a central role in the proliferation, migration, and collagen deposition of airway smooth muscle cells (Johnson et al. 2022; Khan et al. 2013; Mahn et al. 2010), in which both the Gβγ subunit and Epac (exchange protein directly activated by cAMP) play key roles in regulating intracellular Ca^2+^ signaling and airway remodeling (Chen et al. 2019; Fuentes et al. 2021). Upon activation of certain GPCRs, the dissociated Gβγ dimer can directly modulate voltage‐gated calcium channels (Dolphin 2003) (51) and transient receptor potential (TRP) channels (Gaidin et al. 2020; Zhao and MacKinnon 2023). Meanwhile, Epac, as a direct effector of cAMP, activates Rap1 in a PKA‐independent manner (Doebele et al. 2009; Young et al. 2019), thereby influencing Ca^2+^ mobilization from intracellular stores and Ca^2+^ influx (Kosuru et al. 2024). The crosstalk between Gβγ‐ and Epac‐mediated signaling pathways at the level of Ca^2+^ homeostasis finely tunes the proliferation, migration, and contraction of airway smooth muscle cells. Although the present study primarily focused on the ADORA2A–cAMP–PKA–NLRP3 inflammatory axis, the upregulation of ADORA2A expression by PCN may also modulate the Gβγ and Epac signaling branches, thereby regulating Ca^2+^ signaling and subsequently alleviating airway remodeling. This hypothesis is supported by the following evidence: KEGG enrichment analysis clearly identified the calcium signaling pathway as significantly enriched, and lung histopathological damage (e.g., alveolar collapse and bronchial stenosis) was markedly ameliorated following PCN treatment. Whether PCN‐mediated ADORA2A upregulation engages parallel pathways—such as cAMP‐Epac signaling or Gβγ‐mediated calcium modulation—to alleviate airway remodeling warrants further investigation.

Although the present study systematically elucidated the pharmacodynamic material basis and molecular mechanisms of PCN in the treatment of asthma, several limitations should be acknowledged. ADORA2A was identified as the core target solely through network pharmacology and molecular docking, and its functional role has not been rigorously validated using gene knockout or specific antagonists/agonists. The crosstalk between ADORA2A signaling and the Gβγ‐Epac‐Ca^2+^ pathway is only preliminarily hypothesized, and no mechanistic rescue experiments have been performed to confirm this interaction. The individual effects and synergistic interactions of core absorbed components such as 5‐*O*‐methylvisammioside and nobiletin remain unclear. Moreover, the pharmacokinetic profiles and tissue distribution of the absorbed components have not been systematically investigated. Future studies should focus on target‐specific validation, elucidation of signaling pathway crosstalk, and screening of core pharmacodynamic components to further refine the mechanisms underlying the therapeutic effects of PCN in asthma.

## 5 Conclusions

In this study, 107 compounds were identified from PCN, 36 of which were detected in the serum of asthmatic rats. Network pharmacology and molecular docking identified ADORA2A as the key therapeutic target, with four compounds (5‐*O*‐methylvisammioside, nobiletin, *sec*‐*O*‐glucosylhamaudol, and 9‐oxo‐ODE) showing moderate binding affinity. In vivo experiments confirmed that PCN restored body weight, alleviated airway hyperresponsiveness and lung tissue damage, reduced inflammatory cytokine levels, upregulated ADORA2A expression, elevated cAMP levels, increased PKA phosphorylation, and downregulated NLRP3 expression. These findings preliminarily elucidate the pharmacodynamic material basis and potential mechanism of PCN in treating asthma, laying a foundation for further development of PCN.

This study also has certain limitations: The key target ADORA2A was identified mainly through network pharmacology and molecular docking, but no target knockout or specific inhibitor was used to confirm its necessity in vivo. Moreover, although four compounds showed strong binding affinity to ADORA2A, their individual or synergistic contributions to the therapeutic effects of PCN were not experimentally validated. Additionally, the pharmacokinetic profiles of the 36 absorbed compounds, including their actual concentrations and metabolic stability in serum, were not determined. Finally, the study focused on the ADORA2A/cAMP/PKA/NLRP3 pathway, while other possible signaling pathways or off‐target effects of PCN were not explored. We will further improve these aspects in subsequent experiments.

## Author Contributions

Z.Y. designed the study, performed the experiments, and wrote the manuscript. D.Y.W. performed the experiments, processed the data, and assisted in writing the manuscript. B.Y.B. performed the network pharmacology analysis. Y.E.D. provided assistance with the animal experiments. C.W.D. guided the analysis of the PCN components. W.Y.Y. designed the study and helped revise the manuscript. F.X.M. designed the study and provided funding support.

## Funding

This work was supported by the Postdoctoral Research Project of the Department of Human Resources and Social Security of Anhui Province (Wan Ren She Mi [2024] No. 231) and National Natural Science Foundation of China (General Program, Grant No. 82575020).

## Disclosure

All claims expressed in this article are solely those of the authors and do not necessarily represent those of their affiliated organizations or those of the publisher, the editors, and the reviewers. Any product that may be evaluated in this article, or claim that may be made by its manufacturer, is not guaranteed or endorsed by the publisher.

## Ethics Statement

This study was reviewed and approved by the Animal Ethics Committee of Anhui University of Chinese Medicine, under approval number AHUCM‐rats‐2024108. At the conclusion of the experiment, all animal carcasses were uniformly transported to the animal facility for incineration.

## Conflicts of Interest

The authors declare no conflicts of interest.

## Supporting information


**Table S1:** Primer sequences for RT‐qPCR.
**Table S2:** UHPLC‐Q‐Orbitrap MS analysis results of chemical constituents in PCN.

## Data Availability

The data that support the findings of this study are available on request from the corresponding author. The data are not publicly available due to privacy or ethical restrictions.
