# Supplementary material for: Mechanism of Pingchuanning in Attenuating Asthma Based on UHPLC‐Q‐Orbitrap HRMS and Network Pharmacology Analysis
Source: Biomed Chromatogr. 2026 Oct 19;40(12):e70607. doi: 10.1002/bmc.70607 (PMC13680050; doi:10.1002/bmc.70607)
Supplement: Supplementary file 1 [file BMC-40-e70607-s001.docx]

**Supporting Information**

**Mechanism of Pingchuanning in Attenuating Asthma Based on UHPLC-Q-Orbitrap HRMS and Network Pharmacology Analysis**

Yue Zhang^1,2†^, Yiwen Ding^1,3†^, Weidong Chen^1,3^, Yuanbo Bi^1,3^,

Yanyan Wang^1,3*^, Weidong Ye^1,2,4*^, Xiangming Fang^1,2,4*^

^1^Anhui University of Chinese Medicine, Hefei , China

^2^ School of Traditional Chinese Medicine, Anhui University of Chinese Medicine, Hefei, China

^3^ School of Pharmacy, Anhui University of Chinese Medicine, Hefei, China

^4^Anhui University of Chinese MedicineKey Laboratory of Xin'an Medicine, Ministry of Education, Hefei, China

*CORRESPONDENCE

E-mail addresses: [wangyanyan@ahtcm.edu.cn](mailto:wangyanyan@ahtcm.edu.cn) (Yanyan Wang)

E-mail addresses: [yeweidong@ahtcm.edu.cn](mailto:yeweidong@ahtcm.edu.cn) (Weidong Ye)

E-mail addresses: fxm.bsh@163.com (Xiangming Fang)

†These authors have contributed equally to this work

**Table S1 Primers sequences for RT-qPCR.**

| **Gene** | **Foward Primer** | **Reverse Primer** |
| --- | --- | --- |
| IL-4 | ATGTACCAGGAGCCATATCCACGG | TCCCTTCTCCTGTGACCTCGTT |
| IL-5 | TGGGCCATTGCTATGGAGAT | GCCTCATCGTCTCATTGCTC |
| IL-18 | TCAGACCACTTTGGCAGACT | GAACACAGGCGGGTTTCTTT |
| IL-1β | AATCTCACAGCAGCATCTCGACAAG | TCCACGGGCAAGACATAGGTAGC |
| β-actin | ACATCCGTAAAGACCTCTATGCC | TACTCCTGCTTGCTGATCCAC |

**Table S2 UHPLC-Q-Orbitrap MS analysis results of chemical constituents in PCN**

| Peak | Name | RT [min] | Molecular  formula | Error (ppm) | m/z | Reference ions | Fragment ions (m/z) |
| --- | --- | --- | --- | --- | --- | --- | --- |
| 1 | 0.092 | Asparagine | C4 H8 N2 O3 | -0.06 | 133.06076 | [M+H]+1 | 74.0249,87.0554,70.0303,133.0612,46.0294 |
| 2 | 0.153 | Leucine | C6 H13 N O2 | 0.53 | 132.10197 | [M+H]+1 | 86.0961,69.0693,73.0633,73.0266,97.0615 |
| 3 | 0.748 | Laurolactam | C12 H23 N O | -0.69 | 198.1851 | [M+H]+1 | 198.1852,83.0855,97.1012,69.0698,55.0543 |
| 4 | 1.055 | L-Histidine | C6 H9 N3 O2 | -0.4 | 156.07669 | [M+H]+1 | 156.0765,110.0711,95.0602 |
| 5 | 1.156 | DL-Homoserine | C4 H9 N O3 | 1.33 | 118.05112 | [M-H]-1 | 118.0512,100.0405,72.0456,99.0089,55.0190 |
| 6 | 1.171 | L-Aspartic acid | C4 H7 N O4 | 0.85 | 132.03032 | [M-H]-1 | 88.0387,116.0336,134.0455,70.0282,71.0118 |
| 7 | 1.197 | L-Glutamic acid | C5 H9 N O4 | -0.27 | 148.06039 | [M+H]+1 | 84.0439,102.0569,85.0297 |
| 8 | 1.219 | α,α-Trehalose | C12 H22 O11 | 0.12 | 341.10892 | [M-H]-1 | 341.1000,340.4650,89.5270 |
| 9 | 1.244 | D-(-)-Quinic acid | C7 H12 O6 | 0.77 | 191.05626 | [M-H]-1 | 191.2999,85.2480 |
| 10 | 1.279 | Succinic semialdehyde | C4 H6 O3 | 1.69 | 101.02459 | [M-H]-1 | 101.0246,83.0141,73.0297 |
| 11 | 1.284 | (2R)-2,3-Dihydroxypropanoic acid | C3 H6 O4 | 1.45 | 105.01949 | [M-H]-1 | 105.0999,74.9844,56.9434,59.2325 |
| 12 | 1.284 | Cytosine | C4 H5 N3 O | -0.9 | 112.05044 | [M+H]+1 | 95.0231,112.0484,94.0387,52.0179,68.0123 |
| 13 | 1.306 | Adenine | C5 H5 N5 | -0.82 | 136.06166 | [M+H]+1 | 136.0612,137.0640,119.0350 |
| 14 | 1.329 | Itaconic acid | C5 H6 O4 | 1.13 | 175.02496 | [M+FA-H]-1 | 85.1999,129.1113,83.2800 |
| 15 | 1.346 | Ephedrine | C10 H15 N O | 0.84 | 166.12276 | [M+H]+1 | 166.1227,148.1121,117.0702 |

**(continuous) TableS2 UHPLC-Q-Orbitrap MS analysis results of chemical constituents in PCN**

| Peak | Name | RT [min] | Molecular  formula | Error (ppm) | m/z | Reference ions | Fragment ions (m/z) |
| --- | --- | --- | --- | --- | --- | --- | --- |
| 16 | 1.39 | Isocitric acid | C6 H8 O7 | 0.81 | 191.01988 | [M-H]-1 | 111.2390,85.2166,155.1101  116.9900 |
| 17 | 1.393 | DL-Malic acid | C4 H6 O5 | 0.96 | 133.01437 | [M-H]-1 | 114.9999,71.4310,133.2230 |
| 18 | 1.408 | Nicotinamide | C6 H6 N2 O | -0.37 | 123.05524 | [M+H]+1 | 123.0553 80.0489 96.0446,106.0282 |
| 19 | 1.431 | Phenylephrine | C9 H13 N O2 | -0.59 | 168.10181 | [M+H]+1 | 91.0559,150.0925,107.0494,135.0681,134.0616 |
| 20 | 1.447 | Nicotinic acid | C6 H5 N O2 | -0.03 | 124.03931 | [M+H]+1 | 124.0396,78.0343 |
| 21 | 1.462 | Malonic acid | C3 H4 O4 | 1.5 | 103.00384 | [M-H]-1 | 103.0999,75.3140,102.1100,57.3100 |
| 22 | 1.464 | 2-Oxoglutaric acid | C5 H6 O5 | 1.07 | 191.01988 | [M+FA-H]-1 | 100.8999,57.5520,144.6125.73.4960,99.7100 |
| 23 | 1.472 | Guanine | C5 H5 N5 O | -0.61 | 152.05659 | [M+H]+1 | 152.0553,135.0297,110.0353,55.0301,80.0236 |
| 24 | 1.525 | Fumaric acid | C4 H4 O4 | 1.14 | 115.00381 | [M-H]-1 | 70.8821,114.7478,45.2072,114.1467 |
| 25 | 1.545 | DL-Arginine | C6 H14 N4 O2 | -0.15 | 175.11893 | [M+H]+1 | 70.0669,116.0737,60.0566 |
| 26 | 1.55 | D-(+)-Mannose | C6 H12 O6 | 1.34 | 179.05634 | [M-H]-1 | 59.0137,89.0244,71.0138,101.0244,119.0349 |
| 27 | 1.865 | cis-Aconitic acid | C6 H6 O6 | 0.55 | 173.00925 | [M-H]-1 | 85.0278,129.0189 |
| 28 | 1.865 | Citric acid | C6 H8 O7 | 0.58 | 191.01984 | [M-H]-1 | 41.0032,57.0341 |
| 29 | 1.932 | Adenosine | C10 H13 N5 O4 | -0.9 | 268.10379 | [M+H]+1 | 136.0615,57.0335,119.0343 |
| 30 | 1.934 | 4-Hydroxybenzaldehyde | C7 H6 O2 | -0.53 | 123.04399 | [M+H]+1 | 123.0439,95.0493,77.0386 |
| 31 | 1.947 | Adenine | C5 H5 N5 | 1.41 | 134.04741 | [M-H]-1 | 107.0367.134.0481.92.0251,65.0143,41.0233 |
| 32 | 1.953 | trans-Aconitic acid | C6 H6 O6 | 1.02 | 173.00934 | [M-H]-1 | 85.0295,129.0192,111.0087,173.0089 |

**(continuous) TableS2 UHPLC-Q-Orbitrap MS analysis results of chemical constituents in PCN**

| Peak | Name | RT [min] | Molecular  formula | Error (ppm) | m/z | Reference ions | Fragment ions (m/z) |
| --- | --- | --- | --- | --- | --- | --- | --- |
| 33 | 2.092 | L-Norleucine | C6 H13 N O2 | -0.29 | 132.10187 | [M+H]+1 | 86.0321,131.9420,69.1192 |
| 34 | 2.123 | Succinic acid | C4 H6 O4 | 1.24 | 117.01947 | [M-H]-1 | 117.0194,73.0295,99.0088 |
| 35 | 2.175 | 3-Hydroxy-3-methylglutaric acid | C6 H10 O5 | 1.32 | 161.04576 | [M-H]-1 | 101.2443,57.3352,161.3147,81.2130 |
| 36 | 2.433 | Hordenine | C10 H15 N O | -0.4 | 166.12257 | [M+H]+1 | 121.0652,77.0390,103.0546 |
| 37 | 2.542 | 3-Hydroxybutyric acid | C4 H8 O3 | 2 | 103.04028 | [M-H]-1 | 103.0395,59.0141 |
| 38 | 2.751 | Gallic acid | C7 H6 O5 | 1.56 | 169.01451 | [M-H]-1 | 169.1310,81.2230,97.3230,79.3140 |
| 39 | 2.952 | N6-Me-Adenosine | C11 H15 N5 O4 | 0.36 | 282.11978 | [M+H]+1 | 150.0754,282.1189 |
| 40 | 3.012 | Salicylic acid | C7 H6 O3 | 1.3 | 137.0246 | [M-H]-1 | 93.0331,65.0384,137.0231 |
| 41 | 4.087 | Citraconic acid | C5 H6 O4 | 1.1f8 | 129.01949 | [M-H]-1 | 85.0295,129.0193 |
| 42 | 4.255 | Vanillic acid | C8 H8 O4 | 0.76 | 167.03511 | [M-H]-1 | 167.0350,357.0583,152.0109 |
| 43 | 4.262 | Pantothenic acid | C9 H17 N O5 | 0.76 | 218.10356 | [M-H]-1 | 218.3999,146.4500,114.7300,185.1200 |
| 44 | 4.445 | Methylsuccinic acid | C5 H8 O4 | 0.63 | 131.03506 | [M-H]-1 | 87.0453,131.0345 |
| 45 | 4.474 | 2,4-Dihydroxybenzoic acid | C7 H6 O4 | 0.6 | 153.01942 | [M-H]-1 | 153.0194,191.1145,283.8647,109.0296,285.0262 |
| 46 | 4.475 | 2-hydroxy-5-[3,4,5-trihydroxy-6-(hydroxymethyl)oxan-2-yl]oxybenzoic acid | C13 H16 O9 | 0.83 | 315.07242 | [M-H]-1 | 315.0742,315.0571 |
| 47 | 4.63 | Glycyl-L-leucine | C8 H16 N2 O3 | -0.26 | 189.12332 | [M+H]+1 | 86.1121,131.9424,44.3913,43.3762 |
| 48 | 5.507 | Proline | C5 H9 N O2 | -0.22 | 116.07058 | [M+H]+1 | 70.0677,43.0571,74.0981,116.0736,68.0608 |
| 49 | 5.752 | (-)-Ephedrine | C10 H15 N O | -1.24 | 166.12244 | [M+H]+1 | 166.1227,148.1121,117.0702 |
| 50 | 5.795 | Protocatechuic acid | C7 H6 O4 | 1.19 | 153.01952 | [M-H]-1 | 109.0297,153.0188,108.0218 |
| 51 | 6.173 | 5-Sulfosalicylic acid | C7 H6 O6 S | 1.33 | 216.98152 | [M-H]-1 | 198.9706,137.0243,216.9812 |
| 52 | 6.296 | 3-Furoic acid | C5 H4 O3 | 1.35 | 111.00892 | [M-H]-1 | 49.0094,41.0035,39.0236,65.0027,104.1151 |
| 53 | 7.597 | 1,5-Naphthalenediamine | C10 H10 N2 | 0.48 | 159.09175 | [M+H]+1 | 159.0919,143.0733 |

**(continuous) TableS2 UHPLC-Q-Orbitrap MS analysis results of chemical constituents in PCN**

| Peak | Name | RT [min] | Molecular  formula | Error (ppm) | m/z | Reference ions | Fragment ions (m/z) |
| --- | --- | --- | --- | --- | --- | --- | --- |
| 54 | 7.653 | Indole-3-acrylic acid | C11 H9 N O2 | -0.42 | 188.07053 | [M+H]+1 | 170.0630,188.0741,171.0671,189.0766,44.0506 |
| 55 | 7.797 | DL-4-Hydroxyphenyllactic acid | C9 H10 O4 | 0.99 | 181.05081 | [M-H]-1 | 181.0501,163.0398,134.0372 |
| 56 | 8.105 | α-Aspartylphenylalanine | C13 H16 N2 O5 | -0.03 | 281.11319 | [M+H]+1 | 263.0100.235.1303,245.1252,246.1199,166.1147 |
| 57 | 8.461 | Pyrrole-2-carboxylic acid | C5 H5 N O2 | 1.7 | 110.02494 | [M-H]-1 | 109.9999,65.9118,45.6200,79.3100 |
| 58 | 9.002 | 2,5-Dihydroxybenzaldehyde | C7 H6 O3 | 0.97 | 137.02455 | [M-H]-1 | 109.0283,108.0205,137.0234,81.0334,95.0129 |
| 59 | 9.573 | 2-Isopropylmalic acid | C7 H12 O5 | 1.51 | 175.06146 | [M-H]-1 | 115.4210,128.8500,113.2400,157.3400 |
| 60 | 9.615 | 2-Hydroxyphenylacetic acid | C8 H8 O3 | 1.52 | 151.0403 | [M-H]-1 | 107.0507,151.0412,76.0636 |
| 61 | 9.757 | 2-Furoic acid | C5 H4 O3 | 1.76 | 111.00896 | [M-H]-1 | 67.0192,53.9398,53.9171 |
| 62 | 9.815 | 3-Methylglutaric acid | C6 H10 O4 | 1.12 | 145.0508 | [M-H]-1 | 101.0607,127.0394 |
| 63 | 10.91 | (-)-Epigallocatechin | C15 H14 O7 | -0.67 | 307.08102 | [M+H]+1 | 139.1009,151.1156,289.0032,181.1483,169.1591 |
| 64 | 11.534 | Pyrogallol | C6 H6 O3 | -0.42 | 127.03892 | [M+H]+1 | 109.0284,81.0335,127.0390,53.0389 |
| 65 | 11.842 | trans-Cinnamaldehyde | C9 H8 O | 0.08 | 133.0648 | [M+H]+1 | 133.064,105.0701,133.1010,134.0679 |
| 66 | 11.953 | Chlorogenic acid | C16 H18 O9 | 0.84 | 353.0881 | [M-H]-1 | 191.1000,190.2229,189.3420 |
| 67 | 12.005 | Isophthalic acid | C8 H6 O4 | 1.38 | 165.01956 | [M-H]-1 | 121.0295,165.0190,93.0346,75.0239,108.0217 |
| 68 | 12.078 | Vanillin | C8 H8 O3 | 1.12 | 151.04024 | [M-H]-1 | 136.0166,108.0218,107.0504,123.0453 |
| 69 | 12.375 | 2-(2-amino-3-methylbutanamido)-3-phenylpropanoic acid | C14 H20 N2 O3 | -0.25 | 265.1546 | [M+H]+1 | 166.0873,72.0813,166.0697,120.0867 |
| 70 | 12.756 | Sinapinic acid | C11 H12 O5 | -0.64 | 207.06503 | [M+H]+1 | 225.0753,208.0682,175.0385,183.0651 |

**(continuous) TableS2 UHPLC-Q-Orbitrap MS analysis results of chemical constituents in PCN**

| Peak | Name | RT [min] | Molecular  formula | Error (ppm) | m/z | Reference ions | Fragment ions (m/z) |
| --- | --- | --- | --- | --- | --- | --- | --- |
| 71 | 13.003 | D(-)-Amygdalin | C20 H27 N O11 | 1.04 | 502.15714 | [M+FA-H]-1 | 323.2000 ,456.1511,179.1000,221.1000 |
| 72 | 13.43 | 2-Hydroxycaproic acid | C6 H12 O3 | 1.62 | 131.07158 | [M-H]-1 | 85.2100,113.0274,129.3200,83.2157 |
| 73 | 13.639 | Neochlorogenic acid | C16 H18 O9 | 1.36 | 353.08829 | [M-H]-1 | 191.1000,84.6460,92.6140,58.5120 |
| 74 | 13.845 | 3,3-Dimethylglutaric acid | C7 H12 O4 | 1.63 | 159.06654 | [M-H]-1 | 159.0663,115.2000,141.2000,159.2000 |
| 75 | 14.579 | Ferulic acid | C10 H10 O4 | -0.58 | 177.0545 | [M+H]+1 | 116.0100,88.0990,89.0555,117.0412 |
| 76 | 15.156 | Coumarin | C9 H6 O2 | -0.44 | 147.04399 | [M+H]+1 | 147.0443,148.0476,150.1126,103.0543 |
| 77 | 15.33 | Ferulic acid | C10 H10 O4 | 1.4 | 193.0509 | [M-H]-1 | 133.0289,114.1446,160.8440,105.0313,118.9376 |
| 78 | 15.478 | Naringenin | C15 H12 O5 | -0.36 | 273.07565 | [M+H]+1 | 153.0169,91.0533,119.0492,147.0427,154.0258 |
| 79 | 16.507 | {(1R,2R)-2-[(2Z)-5-(Hexopyranosyloxy)-2-penten-1-yl]-3-oxocyclopentyl}acetic acid | C18 H28 O9 | 1.39 | 387.1666 | [M-H]-1 | 387.1645,163.1101,119.0349,163.1019,101.0199 |
| 80 | 17.191 | 1-(Carboxymethyl)cyclohexanecarboxylic acid | C9 H14 O4 | 1.29 | 185.08218 | [M-H]-1 | 141.0918,185.0829 |
| 81 | 17.586 | Catechin | C15 H14 O6 | 1.74 | 289.07227 | [M-H]-1 | 109.0100,108.0506,124.0422,123.0294,122.0270 |
| 82 | 17.628 | Catechin | C15 H14 O6 | 0.17 | 291.08636 | [M+H]+1 | 139.0382,207.0652,165.0537,123.0438,147.0431 |
| 83 | 18.142 | Riboflavin | C17 H20 N4 O6 | 2.14 | 375.13177 | [M-H]-1 | 255.0893,241.0743,212.0854,41.9987 |
| 84 | 18.913 | Methyl cinnamate | C10 H10 O2 | -0.33 | 163.0753 | [M+H]+1 | 131.0491,89.0596,121.0647,107.0701,103.0542 |
| 85 | 19.255 | 3-Phenyllactic acid | C9 H10 O3 | 1.02 | 165.05589 | [M-H]-1 | 165.1999,147.2710,59.1900,111.2500, |
| 86 | 19.857 | 2-Hydroxy-1-(4-methoxyphenyl)propyl hexopyranoside | C16 H24 O8 | 1.21 | 343.14026 | [M-H]-1 | 59.0127,80.9643,89.0232,78.9848,71.0131 |

**(continuous) TableS2 UHPLC-Q-Orbitrap MS analysis results of chemical constituents in PCN**

| Peak | Name | RT [min] | Molecular  formula | Error (ppm) | m/z | Reference ions | Fragment ions (m/z) |
| --- | --- | --- | --- | --- | --- | --- | --- |
| 87 | 19.931 | 3-Coumaric acid | C9 H8 O3 | 1.59 | 163.04033 | [M-H]-1 | 119.0498,91.0542,117.7054,117.0296 |
| 88 | 20.133 | N-Acetyl-L-phenylalanine | C11 H13 N O3 | 1.44 | 206.08257 | [M-H]-1 | 91.0563,58.0291,164.0722,147.0455,103.0552 |

| 89 | 21.859 | Suberic acid | C8 H14 O4 | 1.8 | 173.08224 | [M-H]-1 | 83.0921,57.2266,111.0175,109.0352,81.0561 |
| --- | --- | --- | --- | --- | --- | --- | --- |
| 90 | 21.895 | Pilocarpine | C11 H16 N2 O2 | 0.19 | 209.12849 | [M+H]+1 | 209.1284,163.1238,95.0604,96.068 |
| 91 | 22.76 | (1ξ)-1,5-Anhydro-1-[2-(3,4-dihydroxyphenyl)-5,7-dihydroxy-4-oxo-4H-chromen-8-yl]-D-galactitol | C21 H20 O11 | 1.65 | 447.09402 | [M-H]-1 | 327.0496,447.0928,357.0584,328.0554,297.0394 |
| 92 | 22.783 | (1ξ)-1,5-Anhydro-1-[2-(3,4-dihydroxyphenyl)-5,7-dihydroxy-4-oxo-4H-chromen-8-yl]-D-galactitol | C21 H20 O11 | -0.04 | 449.10782 | [M+H]+1 | 327.0508,447.0938,357.0615,297.0411 |
| 93 | 23.471 | Cyclo(phenylalanyl-prolyl) | C14 H16 N2 O2 | 0.66 | 245.12862 | [M+H]+1 | 154.0742,103.0539,153.0667 |
| 94 | 23.666 | Cathinone | C9 H11 N O | -0.21 | 150.09131 | [M+H]+1 | 132.0000,133.0000,119.0000,150.0913 |
| 95 | 24.194 | 1-(β-D-Glucopyranosyloxy)-7-methyl-1,4a,5,6,7,7a-hexahydrocyclopenta[c]pyran-4-carboxylic acid | C16 H24 O9 | 3.59 | 359.13605 | [M-H]-1 | 197.0795,359.1309,153.0891,135.0804,109.0648 |
| 96 | 24.932 | 2-(acetylamino)-3-(1H-indol-3-yl)propanoic acid | C13 H14 N2 O3 | 1.38 | 245.09351 | [M-H]-1 | 74.0244,116.0499,203.0827 |
| 97 | 24.952 | Cynaroside | C21 H20 O11 | 1.99 | 447.09418 | [M-H]-1 | 285.0499,286.0441,412.8370,287.0472 |
| 98 | 25.218 | 4-Indolecarbaldehyde | C9 H7 N O | 1.57 | 144.04572 | [M-H]-1 | 144.0455,144.2000,116.1000 |
| 99 | 25.344 | Xanthorhamnin | C34 H42 O20 | 2.06 | 769.22125 | [M-H]-1 | 769.2191,315.0503 |
| 100 | 25.703 | Eriocitrin | C27 H32 O15 | 2.19 | 595.16815 | [M-H]-1 | 287.0538,151.0012,595.1644,288.0582,135.0437 |

**(continuous) TableS2 UHPLC-Q-Orbitrap MS analysis results of chemical constituents in PCN**

| Peak | Name | RT [min] | Molecular  formula | Error (ppm) | m/z | Reference ions | Fragment ions (m/z) |
| --- | --- | --- | --- | --- | --- | --- | --- |

| 101 | 26.056 | Vitexin | C21 H20 O10 | 0.62 | 433.11319 | [M+H]+1 | 433.1106,313.0706,283.0608,284.0654 |
| --- | --- | --- | --- | --- | --- | --- | --- |
| 102 | 26.532 | 1,5-Anhydro-1-[5,7-dihydroxy-3-(4-hydroxyphenyl)-4-oxo-4H-chromen-8-yl]hexitol | C21 H20 O10 | 0.06 | 433.11295 | [M+H]+1 | 283.0598,284.0671,79.0184,256.0727,313.0703 |
| 103 | 26.628 | Rutin | C27 H30 O16 | 2.52 | 609.14764 | [M-H]-1 | 609.1472,607.1321 |
| 104 | 26.638 | Quercetin 3-rutinoside | C27 H30 O16 | -0.62 | 611.16028 | [M+H]+1 | 611.0100,303.0171,302.0125,612.0916 |
| 105 | 27.543 | Quercetin-3β-D-glucoside | C21 H20 O12 | 2.56 | 463.08939 | [M-H]-1 | 300.0279,463.0873,255.0282 |
| 106 | 27.561 | Isoquercitrin | C21 H20 O12 | 0.66 | 465.10306 | [M+H]+1 | 463.0867,255.0292 |
| 107 | 28.524 | myricetin 3-O-beta-D-galactopyranoside | C21 H20 O13 | 2.77 | 479.08444 | [M-H]-1 | 316.0225,479.0826,271.0251 |
